# *PHLDA1* silencing in IMR-32 human neuroblastoma cells results in *ABCB1* overexpression, augments chemoresistance and leads to increased growth of tumors

**DOI:** 10.1038/s41598-025-33551-0

**Published:** 2025-12-22

**Authors:** Maja Kudrycka, Irena Horwacik, Anna A. Brożyna, Monika Żywicka, Małgorzata Durbas, Hanna Rokita

**Affiliations:** 1 Laboratory of Molecular Genetics and Virology, Faculty of Biochemistry, Biophysics and Biotechnology, Jagiellonian University, Kraków, Poland; 2 Doctoral School of Exact and Natural Sciences, Jagiellonian University, Kraków, Poland; 3 Department of Human Biology, Institute of Biology, Faculty of Biological and Veterinary Sciences, Nicolaus Copernicus University, Toruń, Poland

**Keywords:** *PHLDA1* silencing, ABCB1, Neuroblastoma, Apoptosis, In vivo tumor growth, Cancer, Molecular biology

## Abstract

PHLDA1 (pleckstrin homology-like domain, family A, member 1) is a multifunctional protein involved in pathophysiological conditions such as cancer. Our previous research revealed changes in the proteome of *PHLDA1*-silenced human neuroblastoma IMR-32 cells. One of the proteins that was increased in *PHLDA1*-silenced cells was the efflux pump ABCB1 (ATP binding cassette subfamily B member 1). In this study, ABCB1 efflux pump upregulation in *PHLDA1*-silenced IMR-32 cells led to increased resistance of the cells to xenobiotic agents. Experiments revealed that xenograft tumors grown in immunocompromised mice from *PHLDA1*-silenced cells presented multiple changes in comparison with tumors grown from control cells. Tumors grown from *PHLDA1*-silenced cells were larger and contained fewer apoptotic cells than control tumors. The blood vessels within the tumors grown from *PHLDA1*-silenced cells displayed increased extravasation and the extracellular matrix of the tumors presented increased collagen content in comparison to that of the control tumors. The results showed that silencing *PHLDA1 in vivo* promoted human neuroblastoma cell survival, decreased the apoptotic potential of the cells, disrupted angiogenesis in developing tumors, and altered collagen network formation.

**Supplementary Information:**

The online version contains supplementary material available at 10.1038/s41598-025-33551-0.

## Introduction

PHLDA1 (pleckstrin homology-like domain, family A, member 1) is a protein that plays a role in many cellular functions, and owing to its ambiguous involvement in various types of cancers, this protein is a frequent subject of research^1^. PHLDA1 is involved in apoptosis and is a confirmed target of the P53 protein^2,3^. PHLDA1 participates in the apoptotic process by inhibiting the PI3K/Akt pathway, leading to caspase activation and the induction of cell death in many human cancer cell lines^4,5^. *PHLDA1* expression may be reduced in certain types of cancer, potentially contributing to uncontrolled cancer cell growth^6^. PHLDA1 might act as a tumor suppressor and is a negative regulator of aurora A kinase in breast cancer^7^, and downregulation of *PHLDA1* mRNA expression is associated with poor prognosis in that cancer^8,9^. Moreover, the pleckstrin homology domain is known for binding membrane phospholipids, which suggests that PHLDA1 may be involved in cellular signaling^10^.

PHLDA1 attracted our interest in previous research. Its expression was upregulated in IMR-32 human neuroblastoma (NB) cells after anti-GD2 14G2a antibody treatment^11^. Another study revealed that IMR-32 cells in which the *PHLDA1* gene was silenced presented increased cellular ATP concentrations and increased mitochondrial potential and were less prone to apoptosis than control cells were^12^. Moreover, the level of a marker of poor prognosis in neuroblastoma, TRKB (tropomyosin receptor kinase B), was increased in *PHLDA1*-silenced cells. We showed that silencing of *PHLDA1* gene led to a significant increase in the expression of *AURKA* (encoding Aurora A kinase), increased phosphorylation of p-Aurora A at Thr288 and Akt at Thr308, and lower levels of cleaved PARP and caspase-3 as well as lower activity of apoptosis-executing caspase 3 and − 7. Thus, the overall results suggested a role of PHLDA1 as a pro-apoptotic protein. In addition, a significant decrease in the mRNA level of the P21 protein (a product of the *CDKN1A* gene, which encodes a cell cycle inhibitor), was noted after *PHLDA1* silencing^12^. By flow cytometry analysis of propidium iodide stained cells, we measured slightly higher percentage of cells in G0/G1 phase for the *PHLDA1*-silenced clones with down-regulation of PHLDA1 (ca. 70%) - as compared to WT and Mock1 cells (ca. 65%) and marginally lower number of cells in S and G2/M phases, as compared to WT and Mock1 cells. Our most recent study on the IMR-32 human neuroblastoma cell line revealed noticeable changes in the global proteome and phosphoproteome after *PHLDA1* silencing, which led to the upregulation of proteins associated with mitochondria. Among the proteins identified in *PHLDA1*-silenced cells, ABCB1 was highly upregulated according to mass spectrometry analysis^13^. ABCB1 (other names: MDR1, P-GP1) is an efflux pump that exports xenobiotics and other harmful substances from cells. It is strongly involved in the multidrug resistance of cancer cells (MDR^14^). In human neuroblastoma, multidrug resistance induced by the activity of various ABC transporters often occurs in the tumors of high-risk patients^15^. ABCB1 and several other ABC transporters are regulated directly by the *MYCN* oncogene, which is a major oncogenic driver in neuroblastoma^16,17^.

In this study, the inverse correlation between PHLDA1 and ABCB1 found in our previous studies via mass spectrometry^13^ was confirmed via RT-qPCR and western blotting. The increased level of ABCB1 after *PHLDA1* silencing led to the induction of xenobiotic resistance in IMR-32 and CHP-134 cells. Our in vivo experiments revealed that xenograft tumors derived from *PHLDA1*-silenced cells were larger and more densely packed than control tumor cells, hemorrhagic, and their collagen network was more abundant than that of tumors grown from control cells. These findings point to a role for PHLDA1 in modulating neuroblastoma tumor growth dynamics, ECM remodeling and chemoresistance development.

## Results

### The levels of transcripts and proteins encoded by the *ABCB1* gene are increased in *PHLDA1*-silenced IMR-32 cells

**Fig. 1:**
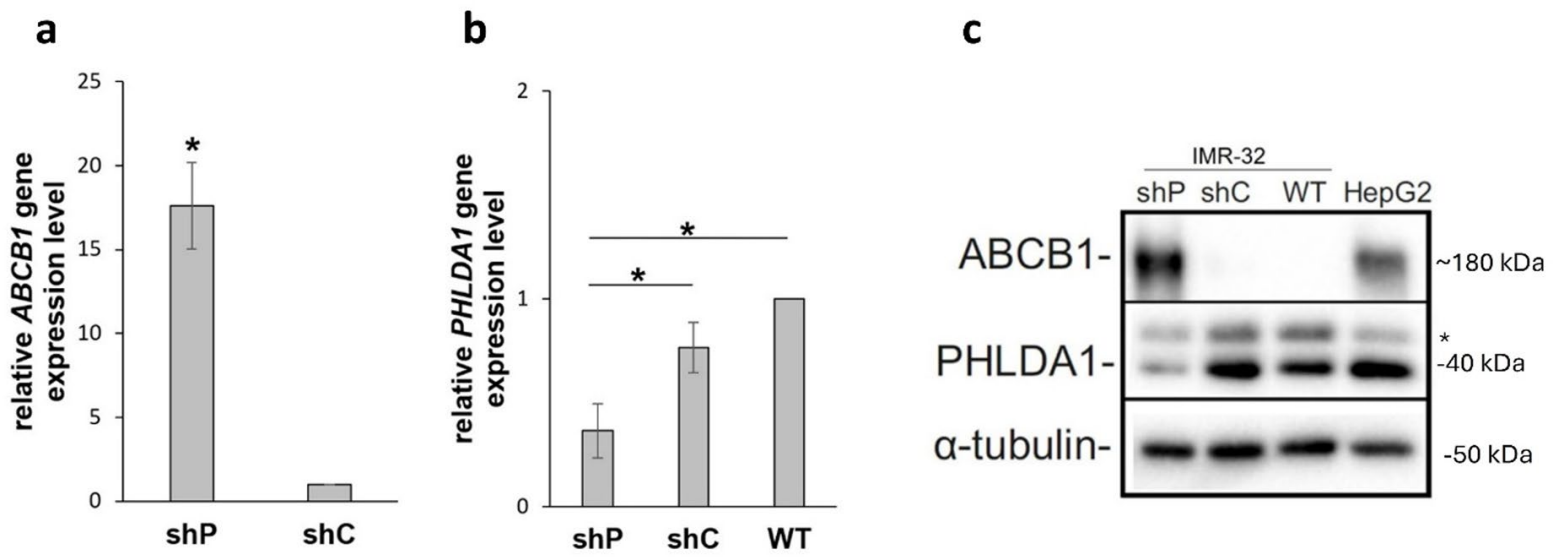
Gene expression analysis of *PHLDA1*-silenced IMR-32 cells (shP), control cells (shC) and wild-type cells (WT) revealed significant changes in *ABCB1* (**a**) and *PHLDA1* (**b**) transcript levels. The *ABCB1* mRNA content in shC was set as 1. The *PHLDA1* mRNA content in the WT was set as 1. The data are shown as the means (± SEM) of 3 (**a**) and 4 (**b**) independent experiments. The samples were run in triplicate. Statistical significance was calculated with Student’s t test for independent samples (*p < 0.05, exact *p* = 0.023) (**a**) and with Kruskal‒Wallis ANOVA followed by Dunn’s test (*p < 0.05, exact *p* = 0.042) (**b**). Western blot analysis of ABCB1 and PHLDA1 in shP, shC and WT IMR-32 cells (**c**). The HepG2 cell line was used as a positive control for the ABCB1 protein. α-Tubulin was used as a reference. Representative immunoblots of 3 independent experiments are shown (*- unspecific band).

Our previous study involving global mass spectrometry analysis revealed that the ABCB1 protein, encoding an efflux pump, was found as one of the characteristic significantly enhanced proteome compound in *PHLDA1*-silenced IMR-32 cells (shP)^13^. To investigate this finding further, RT‒qPCR and western blot analyses were performed (Fig. 1; Supplementary Fig. S1-S3 online). The level of the ABCB1 transcript was more than 15-fold greater in *PHLDA1*-silenced cells (shP) than in control cells (shC) (Fig. 1a). The relative *PHLDA1* gene expression levels are presented in Fig. 1b. As previously reported, *PHLDA1*-silenced cells are characterized by a significantly decreased level of the PHLDA1 transcript^13^. The decreased level of PHLDA1 protein is shown in Fig. 1c (as detected with the anti-PHLDA1 antibody Sc-23866 from Santa Cruz Biotechnology). The nature of the upper band was verified in a separate experiment with an additional anti-PHLDA1 antibody (ab133654 from Abcam, Supplementary Fig. S4 online). Therefore, the upper band shown was labeled as an unspecific band. Moreover, the level of the ABCB1 protein was easily detected in shP cells, although the protein remained undetectable in control shC and WT cells (Fig. 1c).

The level of ABCB1 following *PHLDA1* silencing was assessed in another human neuroblastoma cell line, CHP-134. PHLDA1 and ABCB1 levels were measured in *PHLDA1*-silenced (the S6 and S17 clones), control (the Mock3 clone) and WT cells via western blotting. The upregulation of ABCB1 following the silencing of *PHLDA1* was also confirmed in CHP-134 cells. The levels of ABCB1 in the S6 and S17 *PHLDA1*-silenced clones were greater than those in the Mock3 and WT clones after 48 h of culture in vitro (Supplementary Fig. S5 online).

### *PHLDA1*-silenced IMR-32 cells gained resistance to xenobiotics

The activity of the ABCB1 protein as an efflux pump was examined via two functional assays: a rhodamine 123 accumulation test and a doxorubicin cytotoxicity test.

**Fig. 2:**
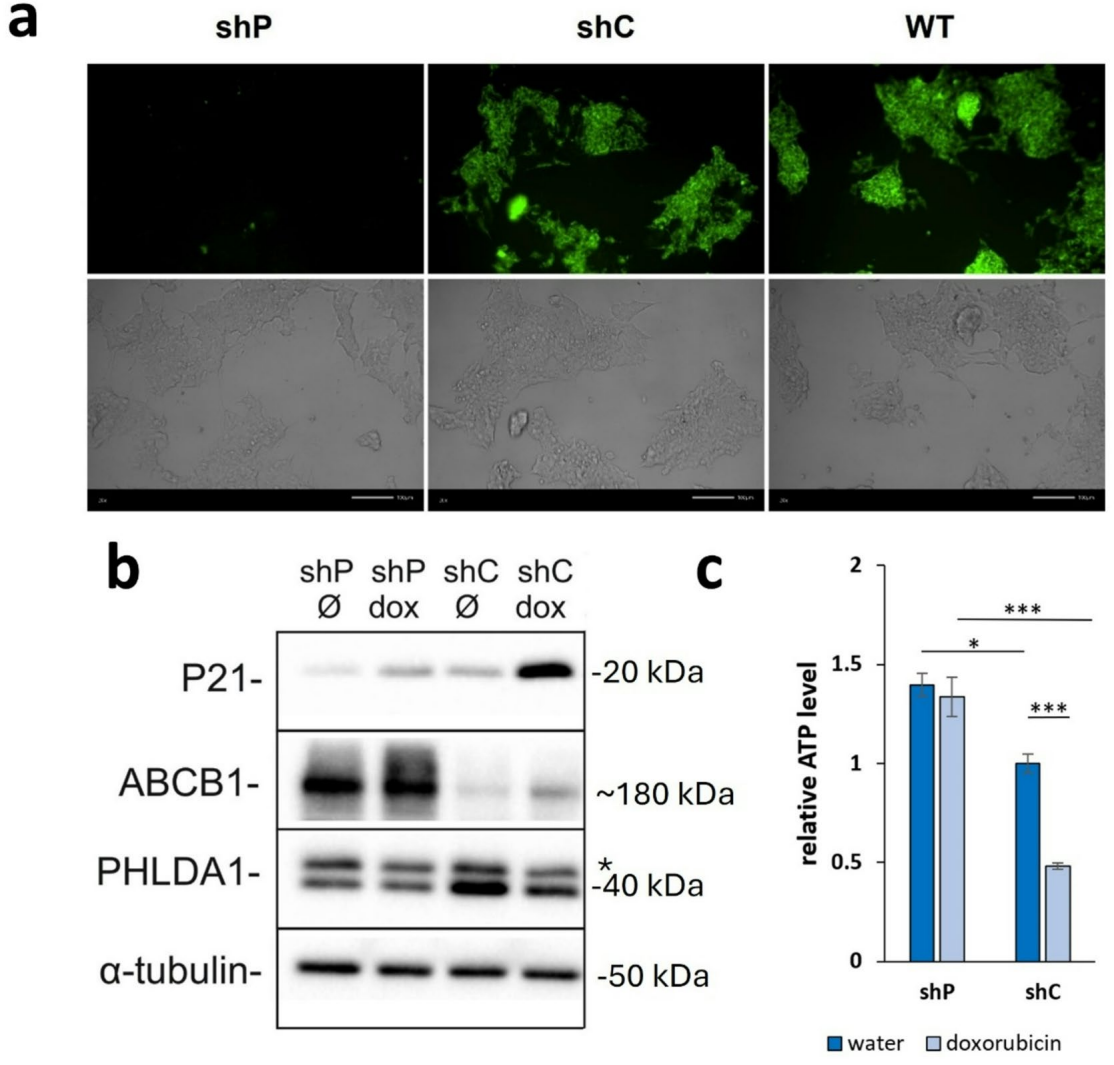
ABCB1 activity in IMR-32 cells with *PHLDA1* silencing. ABCB1 activity was verified via a rhodamine 123 accumulation assay (**a**) and visualized via fluorescence microscopy. Images of *PHLDA1*-silenced (shP), control (shC), and WT IMR-32 cells are shown in fluorescence mode, and visible light is presented in the upper and lower panels, respectively. Scale bars – 100 μm. Fluorescence microphotographs were taken during all three experiments. Brightfield microphotographs were taken during one experiment (Supplementary Fig. S6 online). ABCB1 functional test after doxorubicin treatment (**b**). *PHLDA1*-silenced (shP) and control (shC) IMR-32 cells were treated with 30 nM doxorubicin (dox) or water (Ø) for 48 h, protein lysates were subsequently obtained, and western blot analysis was performed in three independent experiments. Representative immunoblots are shown (*- unspecific band). An ATP luminescence test was performed to confirm the cytotoxic effect of doxorubicin (**c**). The number of control cells treated with water was set to 1. The samples were run in triplicate. The data are shown as the means (± SEM) of three independent experiments. Statistical significance was assessed by one-way ANOVA, followed by post hoc Tukey’s test (*p < 0.05, exact *p* = 0.022 for shP water vs. shC water; ***p < 0.001, exact *p* = 0.00043 for shC water vs. shC doxorubicin; *p* = 0.000024 for shP doxorubicin vs. shC doxorubicin).

Rhodamine 123 is a fluorescent dye that is known to be one of the chemicals actively exported by the ABCB1 protein^18^. To test ABCB1 protein activity in our model, shP, shC and WT cells were treated with 1 µM rhodamine 123 solution for 4 h, and the fluorescence was measured. shC and WT cells displayed high fluorescence intensity because rhodamine 123 accumulated within the cells. In contrast, shP cells were nonfluorescent, i.e., they did not accumulate rhodamine 123. This means that rhodamine 123 was actively and efficiently pumped out of the shP cells (Fig. 2a; Supplementary Fig. S6 online).

Another functional test, an indirect test of ABCB1 protein activity, involved incubating shP and shC cells with doxorubicin (Fig. 2b, c; Supplementary Fig. S7-S10 online). The P21 protein is strongly elevated in many types of cells exposed to doxorubicin^19,20^, including human IMR-32 neuroblastoma cells^21^. Therefore, in this test, the protein served as a marker for the cellular response to this chemotherapeutic agent. *PHLDA1*-silenced cells (shP) and control cells (shC) were incubated with complete medium containing 30 nM doxorubicin (or water as the drug diluent) for 48 h, after which the levels of P21, ABCB1 and PHLDA1 were determined (Fig. 2b). Doxorubicin induced high P21 expression solely in shC cells (Fig. 2b). Additionally, a significant reduction in ATP levels, by more than 50%, was detected in the doxorubicin-treated shC cells, indicating a strong cytotoxic effect of the chemotherapeutic agent in the control IMR-32 cells (Fig. 2c; Supplementary Fig. S10 online). In contrast, the relative ATP levels in shP cells after doxorubicin treatment remained unchanged, indicating that *PHLDA1*-silenced cells gained resistance to doxorubicin. These findings indicate that the ABCB1 efflux pump operates efficiently in *PHLDA1*-silenced cells.

Furthermore, we aimed to investigate the response of CHP-134 cells in which *PHLDA1* was silenced to doxorubicin to examine whether a similar regulatory effect could be observed in yet another neuroblastoma cell line. To assess the cytotoxicity of doxorubicin following *PHLDA1* silencing in CHP134, the intracellular ATP content was again measured. The decreased cytotoxicity of doxorubicin was observed following *PHLDA1* silencing in the S6 and S17 clones of CHP-134 neuroblastoma cells compared with that in the control cells, and a statistically significant decrease in ATP levels was detected only at the highest concentration tested (150 nM) (Supplementary Fig. S11 online).

Finally, IMR-32 cells overexpressing *PHLDA1*, after stable transfection with a plasmid vector, presented lower ABCB1 levels, which correlated with their increased susceptibility to 30 nM doxorubicin (Supplementary Fig. S12, S13 online). Therefore, we can conclude that PHLDA1 affected levels of the ABCB1 transporter in IMR-32 and CHP-134 neuroblastoma cells, which impacted on their sensitivity to doxorubicin.

### Neuroblastoma tumors with the silenced *PHLDA1* gene grew faster than control tumors and displayed abnormalities in blood supply within the tissue

**Fig. 3:**
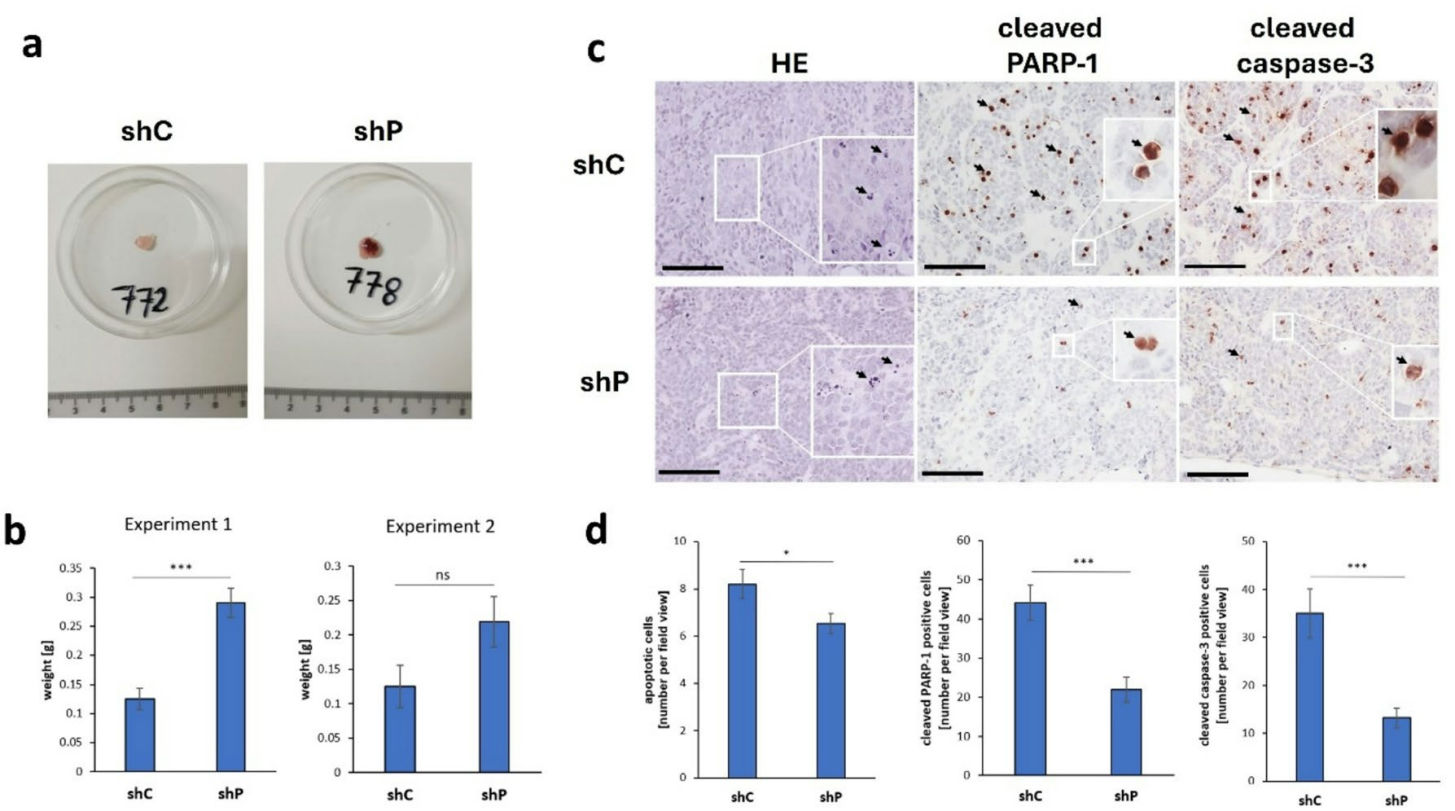
Impact of *PHLDA1* gene silencing on NB tumors grown in vivo in immunocompromised mice. Photographic images of xenograft neuroblastoma tumors grown from *PHLDA1*-silenced (shP) and control cells (shC) in the second experiment (**a**). Photographs of tumors from two complete experiments are presented in Supplementary Fig. S16 online. Comparison of the weights of extracted shC and shP tumors in the first and second experiments (**b**). The data are presented as the means of 8 tumors per group (± SEM). The exact tumor weights are shown in the Supplementary Table S1a, b online. Statistical significance between the groups was measured with two-tailed Student’s t tests for independent samples. ***p < 0.001, exact *p* = 0.00016; ns – not statistically significant. Microscopy images of xenograft neuroblastoma tumors grown from shP and shC cells in immunocompromised mice. The tissue slices were stained with Meyer’s hematoxylin and eosin (left column), cleaved PARP-1 (middle column), and cleaved caspase-3 (right column) (**c**). Enlarged fragments of the images containing apoptotic cells identified in HE-stained sections on the basis of morphological features, cleaved PARP-1- and cleaved caspase-3-positive cells are indicated with white rectangles and are shown as inserts. Scale bars, 100 μm. Mean (± SEM) number of apoptotic cells in the shC and shP groups detected on the basis of morphological features via HE staining, cleaved PARP-1, and cleaved caspase-3 immunostaining, respectively (see Supplementary Table S2a-f online for the counting results from a given tumor sample). Statistical significance was assessed via the two-tailed unpaired Student’s t test for independent samples, *p < 0.05, exact *p* = 0.043 for apoptotic cells; ***p < 0.001, exact *p* = 9.05 × 10^− 14^ for cleaved PARP-1-positive cells; ***p < 0.001, exact *p* = 1.7 × 10^− 12^ for cleaved caspase-3-positive cells (**d**).

**Fig. 4:**
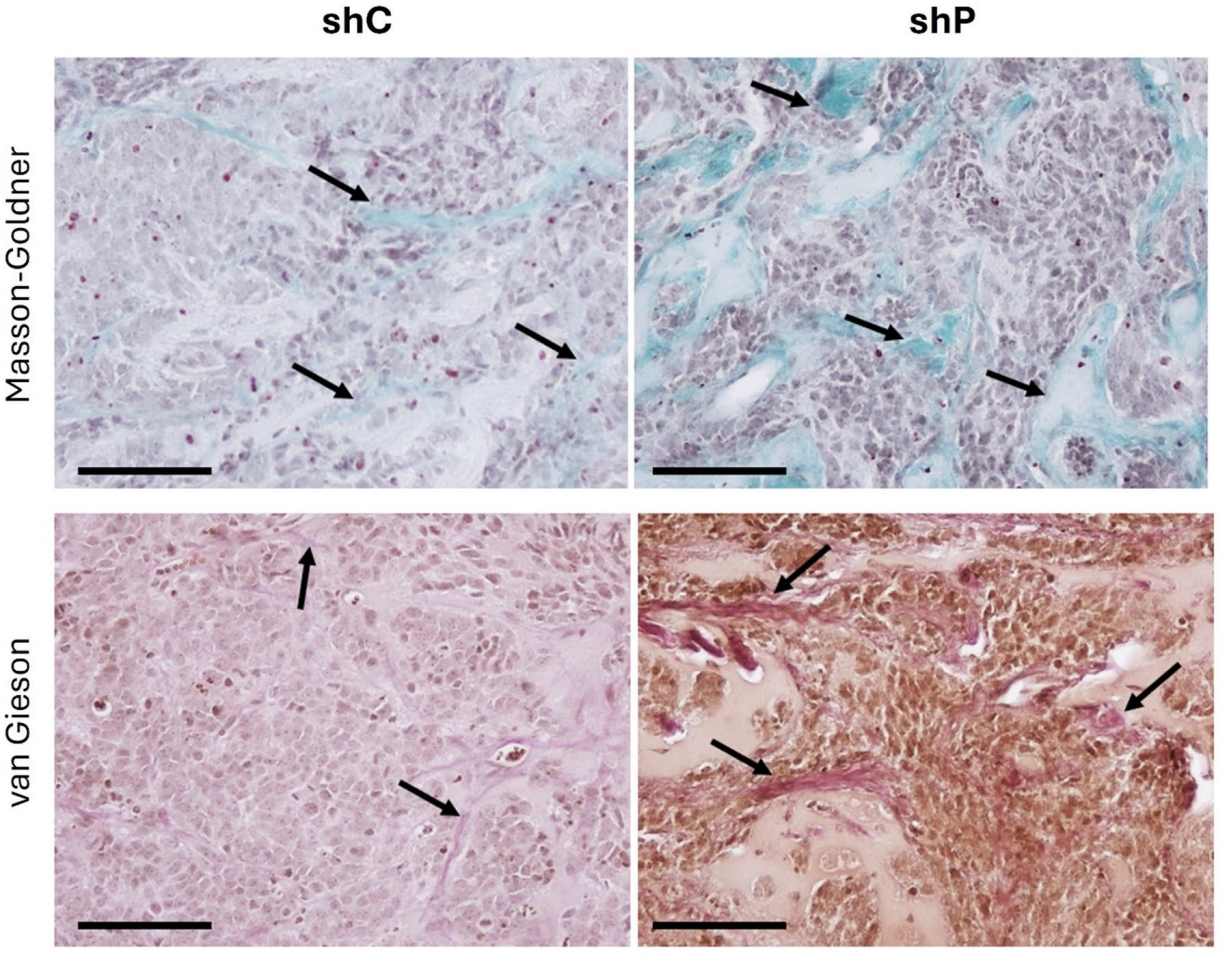
*PHLDA1* silencing augments the collagen net density within tumor tissue. Microscopy images of xenograft neuroblastoma tumors grown from *PHLDA1*-silenced (shP) and control cells (shC) in immunocompromised mice. The tissue slices were stained with Masson-Goldner trichrome or van Gieson trichrome. Scale bars – 100 μm. The arrows indicate collagen fibers stained with Masson-Goldner trichrome or van Gieson trichrome.

Athymic nude mice were injected subcutaneously (s.c.) with shP or shC human neuroblastoma IMR-32 cells to determine the impact of *PHLDA1* silencing on xenograft tumor growth and morphology. The silencing of PHLDA1 was monitored before injecting the cells into the mice (Supplementary Fig. S14, S15 online). Tumors derived from shP cells showed many differences compared to those from shC cells. Macroscopically, tumors derived from shC cells presented light pink coloration, whereas tumors derived from shP cells presented intensely red coloration, indicating a greater blood supply and extravasation (Fig. 3a; Supplementary Fig. S16 online). Indeed, numerous extravasations were observed in tumors derived from shP cells, which were absent in tumors derived from shC cells (Supplementary Fig. S17 online). Another observed difference was that the cells in the shP tumors were packed thicker than those in the control tumors were (Figs. 3c and 4; Supplementary Fig. S17, S18 online). Neuroblastoma tumors with *PHLDA1* silencing tended to have greater masses than tumors derived from control cells did (Fig. 3b; Supplementary Table S1a, b online). This change was clearly visible, although it was statistically significant only for the means of one of the experiments. Microscopic observations revealed that neuroblastoma tumors derived from implanted shP and shC cells display the typical packed, small round cell tumor architecture, which is characteristic of NB tumors (Fig. 3c; Supplementary Fig. S17 online). They exhibited a nonhomogeneous structure. These studies demonstrated that *PHLDA1* silencing significantly affected the morphology of neuroblastoma tumors. The cells within the shP tumors also had more morphologically altered nuclei, indicating frequent mitotic disruptions. Apoptotic cells, identified on the basis of morphological features and the expression of cleaved PARP-1 and cleaved caspase-3, were statistically significantly more common in shC tumors than in shP tumors (Fig. 3c, d; Supplementary Fig. S19, S20 online; Supplementary Table S2a-f online). This finding was consistent with our in vitro observations, where more dead cells were present in the microscopic field of view in the shC group during trypan blue staining for cell counting (for passages and experiments).

### *PHLDA1* silencing led to the accumulation of collagen in neuroblastoma tumors

The xenograft shP tumors were stiff, possibly because of hemorrhages within them and the presence of extracellular matrix (ECM) abnormalities. In that context, the collagen network was examined in shP and shC cells. Masson-Goldner and van Gieson trichrome staining revealed that tumors derived from shP cells contained more collagen fibers and that the fibers were denser than the fibers of tumors derived from shC cells were (Fig. 4; Supplementary Fig. S18 online).

### Expression and localization of PHLDA1 and ABCB1 proteins in tumors grown in immunodeficient mice

**Fig. 5:**
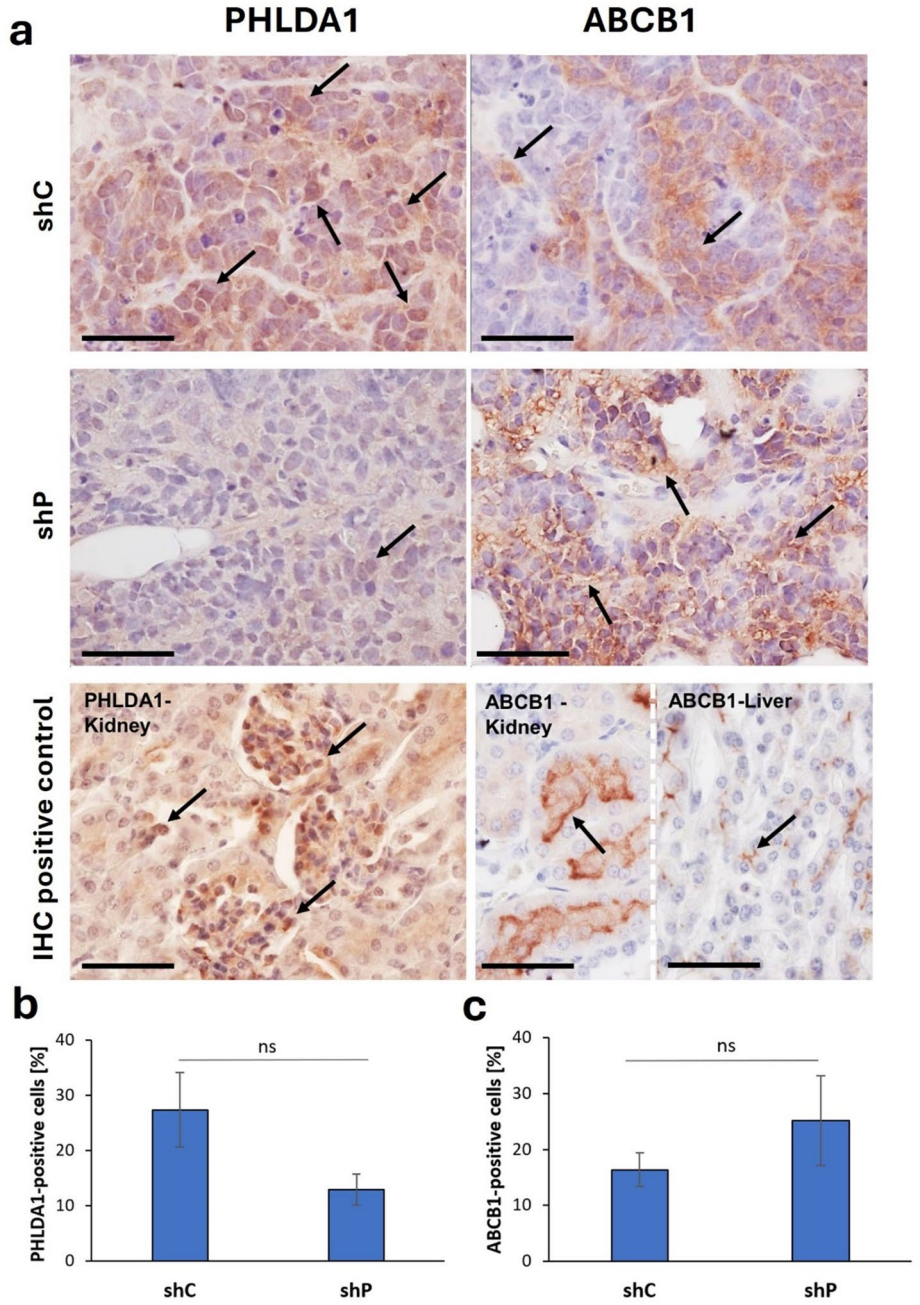
Expression and localization of the PHLDA1 and ABCB1 proteins in tumors grown in vivo from *PHLDA1*-silenced and control human IMR-32 cells (**a**). Representative microphotographs visualizing immunostaining of shC and shP tumors are shown. Nuclei were counterstained with Meyer’s hematoxylin. A positive control for anti-PHLDA1 antibodies was presented in the mouse kidney. A positive control for anti-ABCB1 antibodies was presented on the mouse kidney and mouse liver. Scale bars – 50 μm. Arrows indicate PHLDA1-positive or ABCB1-positive cells, respectively. The percentages of positively stained cells are shown in the graphs below (as means ± SEM, see the Supplementary Table S3 online for the counting results from a given tumor sample). Statistical significance was assessed via the two-tailed unpaired Student’s t test. No statistically significant differences in PHLDA1-positive or ABCB1-positive cell numbers were found between shC and shP tumors (**b, c**).

IMR-32 shP cells used to generate tumors were checked for the occurrence of stable *PHLDA1* silencing via western blot analysis (Supplementary Fig. S14, S15 online). Tumors derived from shP cells presented a lower intensity of immunohistochemical (IHC) staining with anti-PHLDA1 antibodies (Fig. 5a, b; Supplementary Fig. S21 online; Supplementary Table S3 online). After injection and development into tumors in mice, shP cells presented some level of PHLDA1, but the silencing still persisted. In shP slices, the mean number of positively stained cells was lower (13 cells per field view) than that in shC slices (24.8 cells per field view; see Fig. 5a, b; Supplementary Table S3 online); however, statistical significance was not reached. The PHLDA1 level in shP tumors was stably downregulated, compared to tumors derived from shC cells. In shC cells, the staining intensity was greater. In both shP and shC tumors, PHLDA1 localization was cytoplasmic, however in shC tumors localization of the protein was observed as more intense staining foci.

The ABCB1 protein was detected immunohistochemically in both shC and shP tumors (Fig. 5a, c; Supplementary Fig. S22 online; Supplementary Table S3 online), and similar to our previous results, in *PHLDA1*-silenced tumors the level of ABCB1 was increased. However, compared with the results obtained in our in vitro studies, the differences in ABCB1 levels between the groups were not as intense. In shP slices, the mean number of positively stained cells was greater (25.1 cells per field view, Supplementary Table S3 online) than that in shC slices (16 cells per field view, see Fig. 5a, c; Supplementary Table S3 online); however, statistical significance was not reached.

## Discussion

PHLDA1 is a multifunctional protein involved in many cellular pathways, especially the stress response and apoptosis. In recent years, this protein has often been investigated in relation to cancer^1,22^. Our previous study revealed significant upregulation of ABCB1 at the protein level in *PHLDA1*-silenced IMR-32 neuroblastoma cells via mass spectrometry^13^. In the present study, specific transcript and protein levels were measured in *PHLDA1*-silenced IMR-32 cells via RT‒qPCR and western blotting, respectively. The data clearly support our conclusion that the regulation occurs, although indirectly, at the transcriptional level. The explanation of the proposed PHLDA1-ABCB1 association is based on available data from other studies as well as our studies. First, PHLDA1 can bind and negatively regulate the mitotic aurora A kinase in MDA-MB-231 breast cancer cells^7^. Next, we showed^11^ that the induction of PHLDA1 expression by an anti-GD2 ganglioside antibody resulted in decreased levels of aurora A kinase, its activating phosphorylation, and the MYCN oncogene in IMR-32 human neuroblastoma cells. Moreover, aurora A kinase was shown to directly interact with the MYCN protein to sequester this transcription factor and prevent its ubiquitination as well as proteasomal degradation^23^. On the other hand, the *ABCB1* gene is directly transcriptionally activated by MYCN as a transcription factor^16,24^. Therefore, high levels of PHLDA1 can inhibit aurora A kinase, leading to a decrease in MYCN levels followed by diminished stimulation of *ABCB1* gene transcription.

Next, we tested how *PHLDA1* silencing affects neuroblastoma cells in vivo. Our in vivo study provided evidence that the silencing of *PHLDA1* in neuroblastoma cells significantly influenced tumor growth dynamics, vascularization, and extracellular matrix composition in the applied model. The accelerated growth observed in *PHLDA1*-silenced tumors aligned with previous studies that implicated PHLDA1 as a tumor suppressor in various cancers^4^. The presence of numerous extravasations in shP tumors, which were absent in shC tumors, proved that *PHLDA1* silencing may enhance angiogenic processes, leading to a more chaotic and leaky vasculature. This can be clinically relevant, as high levels of angiogenic factors are known to be present in advanced stages of human neuroblastoma^25^. Additionally, in our previous research, we demonstrated that silencing *PHLDA1* led to EGFR overexpression^13^. High levels of EGFR were shown to increase development of blood vessels within various in vivo-generated tumors^26^. Given this information, the upregulation of EGFR could also be one of the reasons for enhanced angiogenesis in shP tumors. Therefore, the inhibition of angiogenesis is a part of the development of therapeutic strategies for treating neuroblastoma^27^. It can be concluded that the aberrant blood supply in shP tumors could amplify rapid tumor growth by providing a more extensive, however disorganized, network for nutrient and oxygen delivery.

Another key finding of this study was the significant change in the ECM composition within *PHLDA1*-silenced tumors, especially in the accumulation of collagen. Changes in the ECM are common in cancers, and increased collagen levels can be related to poor patient prognosis^28,29^. Our analysis of the proteome performed in previously described mass spectrometry experiments^13^ revealed that the PCOLCE and COL6A1 proteins were characteristic of shP cells and were not detected in shC cells. Moreover, bioinformatics analysis performed via tools implemented in R2 (http://r2.amc.nl, http://r2platform.com) with the Versteeg 88 dataset^30^ revealed that high levels of *PCOLCE* mRNA were significantly correlated with decreased overall and relapse-free survival rates in NB patients (Supplementary Fig. S23, S24 online). The increased manifestation of collagen in shP cells observed in histological studies could be correlated with the presence of PCOLCE, which is an important enzyme for ECM reconstruction^31^. These data support our findings from the histological staining analyses of shP tumor xenografts. The increased collagen deposition and collagen net density in shP tumors suggested that PHLDA1 may play a critical role in regulating ECM remodeling in our in vivo neuroblastoma model. This finding is interesting because collagen facilitates metastasis in 3D in vitro models^32^. Moreover, an enhanced collagen network was found in metastatic colorectal cancer, suggesting that some of the collagen genes could be used as biomarkers^33^. A high level of PCOLCE was correlated with poor outcomes in glioma^34^ and gastric cancer^35^. Finally, increases in different types of collagens may also be associated with drug resistance^36^. Our in vivo model was not aimed at studying the metastasis process; therefore, more research is needed to elucidate the correlation between PHLDA1 and the collagen network.

Interestingly, while *PHLDA1* silencing in vitro was associated with highly increased ABCB1 expression, the differences in ABCB1 levels between shP and shC tumors in vivo were less intense. These findings suggest that the tumor microenvironment may modulate the expression of ABCB1 differently than it does in cultured cells or that other compensatory mechanisms, such as ubiquitin-proteasomal degradation of ABCB1^37^, may occur in vivo. More data is needed to elucidate this process.

ABCB1 transporter expression and activity are regulated by several mechanisms. Through expression profiling and chromatin immunoprecipitation studies, MYC oncoproteins were shown to coordinately regulate the transcription of specific ABC transporter genes by acting as either activators or repressors^24^. Additionally, studies have reported an association between high-level ABCB1 expression and poor clinical outcomes and *MYCN* amplification^38^. Furthermore, the implications of ABCB1 regulation in treatment-resistant neuroblastomas have been studied^39^. Rösch et al.. reported that depletion of ABCB1 sensitized neuroblastoma cells to vincristine treatment. Moreover, the analysis of gene expression datasets of more than 50 different neuroblastoma cell lines (primary and relapsed) and more than 160 neuroblastoma patient samples from the pediatric precision medicine platform INFORM (Individualized Therapy For Relapsed Malignancies in Childhood) confirmed a pivotal role of ABCB1 specifically in neuroblastoma resistance at relapse^39^.

With respect to our results, it appears now that PHLDA1 is not the only (although indirect) inhibitor of ABCB1 expression. Possibly, based on our currently presented data and previous studies, during chemo-immunotherapy applied in clinics with anti-GD2 ganglioside antibodies and chemotherapeutics, expression of ABCB1 transporter could be decreased, and neuroblastoma tumors might become more sensitive to chemotherapy. However, verification of this hypothesis requires further investigation. The postulated role of PHLDA1 in the regulation of chemoresistance should be experimentally explored. This is important, as so far, ABCB1 inhibitors have failed in clinical trials, probably because systemic ABCB1 inhibition results in a modified body distribution of its many substrates, including drugs, xenobiotics, and other molecules^40^.

The prognostic value of PHLDA1 was reviewed recently by Durbas in various types of cancer^1^. Decreased expression of PHLDA1 is associated with decreased overall survival in patients with breast cancer, gastric adenocarcinoma, or non-small cell lung cancer. In contrast, increased expression of PHLDA1 is responsible for decreased overall survival in patients with pancreatic adenocarcinoma, oral squamous cell carcinoma, and glioblastoma^1^. Notably, PHLDA1 also plays a role in other than neuroblastoma pediatric tumors, e.g., osteosarcoma, where high PHLDA1 expression is associated with a lower overall survival of osteosarcoma patients^41^. These findings indicate that PHLDA1 has different effects on disease prognosis and overall survival, possibly by acting as either a tumor suppressor or an oncogene, depending on the type of tumor. Therefore, investigating the role of PHLDA1 in tumors is meaningful both experimentally and clinically.

In conclusion, our results help to elucidate the relationship between PHLDA1 and ABCB1 in human neuroblastoma. Our analyses proved that the silencing of *PHLDA1* correlated with the upregulation of ABCB1, which led to increased chemoresistance in shP cells. Moreover, we showed that silencing of *PHLDA1* in vivo caused changes in tumor growth, morphology, vascularization and ECM structure. The upregulation of ABCB1 in *PHLDA1*-silenced cells was not specific to a single cell line. In this work, evidence of a more general mechanism of the induction of ABCB1 following *PHLDA1* silencing was presented in vitro in two neuroblastoma cell lines, i.e., IMR-32 and CHP-134. Further analysis of the regulation between PHLDA1 and efflux pumps (such as ABCB1and ABCC10) is needed in other neuroblastoma cell lines and for treatment with different cytotoxic drugs to establish whether this mechanism operates more broadly. Finally, our results reveal how PHLDA1 affects molecular pathways in both in vitro and in vivo models and provide insight into its role in human neuroblastoma, which can be beneficial for better understanding the tumor biology.

## Methods

### Cell cultures

The IMR-32 human neuroblastoma cell line (CCL-127, ATCC) was cultured in EMEM (M4655, Sigma‒Aldrich) supplemented with 10% FBS (10270106, Gibco), 1% nonessential amino acid solution (NEAA, M7145, Sigma‒Aldrich), 1 mM sodium pyruvate (S8636, Sigma‒Aldrich) and 50 µg/ml gentamicin (G1397, Sigma‒Aldrich). The CHP-134 human neuroblastoma cell line (06122002, ECACC) was cultured in RPMI-1640 (R8758, Sigma‒Aldrich) supplemented with 10% FBS and 50 µg/ml gentamicin. The HepG2 human hepatoma cell line (HB-8065, ATCC) was cultured in high-glucose DMEM (D6429, Sigma‒Aldrich) supplemented with 10% FBS and 50 µg/ml gentamicin. All the cell lines were cultured at 37 °C in an incubator with 5% CO_2_.

### *PHLDA1* silencing

*PHLDA1*-silenced IMR-32 cells (shP) and control cells (shC) were generated previously (for details, see^13)^. shP and shC cells were cultured continuously in EMEM (supplemented with 10% FBS, 1% NEAA, 1 mM sodium pyruvate and 50 µg/ml gentamycin) supplemented with the selective antibiotic 0.5 µg/ml puromycin (sc-108071; Santa Cruz Biotechnology).

For the CHP-134 neuroblastoma cell line, *PHLDA1*-silenced S6 and S17 clones of CHP-134 cells elicited with a lentiviral vector, control (the Mock 3 clone), or WT cells were used^42^. To assess the levels of PHLDA1 and ABCB1 following *PHLDA1* silencing in CHP-134 cells, western blotting was used. For protein isolation, the aforementioned groups of CHP-134 cells (2.5 × 10^5^ per well) were cultured in a 6-well plate for 48 h, harvested and lysed with RIPA buffer.

### *PHLDA1* overexpression

*PHLDA1*-overexpressing IMR-32 cells and control cells were generated via plasmid vector transfection as described in detail previously^13^. A plasmid containing the ORF of *PHLDA1* (Genecopoeia, EX-Z4556-M68) and a control plasmid (Genecopoeia, EX-NEG-M68) were used. Transfected cells were cultured in EMEM (with 10% FBS, 1% NEAA, 1 mM sodium pyruvate and 50 µg/ml gentamycin) supplemented with the selective antibiotic 0.5 µg/ml puromycin (sc-108071; Santa Cruz Biotechnology).

### ATP level measurement

IMR-32 cells (2 × 10^5^ per well) were cultured in 96-well plates for 48 h, and the cells were treated with doxorubicin in triplicate or with water. CHP-134 cells (5 × 10^3^ per well) were treated with doxorubicin at concentrations ranging from 1 to 150 nM or with diluent (water) and cultured in a 96-well plate for 48 h, after which the ATP content was measured in *PHLDA1*-silenced (S6, S17), control (Mock3) and WT cells. The intracellular ATP content was measured via an Infinite M200 Reader (Tecan Group Ltd.) via an ATPlite Luminescence ATP Detection Assay (6016947, PerkinElmer) according to the manufacturer’s protocol.

### Protein isolation and Immunoblotting

To isolate total protein fractions, IMR-32 cells (1 × 10^6^ per well) and CHP-134 cells (2.5 × 10^5^ per well) were cultured in a 6-well plate for 48 h, washed twice in cold PBS and lysed in RIPA buffer supplemented with protease (P8340, Sigma‒Aldrich) and phosphatase inhibitors (PhosSTOP, 4906845001, Roche). The protein concentration was measured with a Bicinchoninic Acid assay (BCA assay, Bicinchoninic Acid Kit for Protein Determination, B9643, C2284, BCA1; Sigma‒Aldrich). Aliquots containing 20–60 µg of total protein lysates were subjected to vertical electrophoresis in polyacrylamide gels via standard SDS‒PAGE procedures and electrotransferred to polyvinylidene difluoride (PVDF) membranes (Millipore Corporation). The obtained blots were blocked with 5% nonfat dry milk in TBS containing 0.1% Tween 20 for 1 h and then incubated overnight at 4 °C with primary antibodies (Table 1). The next day, the membranes were incubated with secondary antibodies conjugated with HRP for 1 h at RT. The immunoreactive bands were detected via a chemiluminescence method (with a luminol reagent, RPN2105 Amersham, Cytiva) on a ChemiDoc device (Bio-Rad laboratories). The membranes were stripped with 0.4 M NaOH and incubated with antibodies against α-tubulin, which was used as a reference protein.

**Table 1:** Antibodies used in this research.

Antigen	Host species	Dilution	Vendor	Catalog no.
ABCB1 (for WB)	Rabbit	1:1,000	Cell Signaling Technology	12683
ABCB1 (for IHC)	Rabbit	1:100	Cell Signaling Technology	13978
P21	Rabbit	1:1,000	Cell Signaling Technology	2947
Cleaved-PARP-1 (for IHC)	Rabbit	1:75	Abcam	ab32064
Cleaved caspase 3 (for IHC)	Rabbit	1:50	R&D Systems, part of Bio-Techne	MAB825-SP
PHLDA1 (for WB)	Mouse	1:1,000	Santa Cruz Biotechnology	Sc-23866
PHLDA1 (for IHC and WB)	Rabbit	1:40 for IHC 1:10,000 for WB	Abcam	ab133654
α-Tubulin	Rabbit	1:4,000	Cell Signaling Technology	2125
Secondary anti-rabbit IgG (for WB)	Goat	1:2,000–1:4,000	Cell Signaling Technology	7074
Secondary anti-rabbit IgG (for IHC)	Goat	Ready-to-use solution	Vector Laboratories	MP-7451
Secondary anti-mouse IgG (for WB)	Horse	1:3,000	Cell Signaling Technology	7076

### RNA isolation and RT‒qPCR

RNA was isolated with TRI-REAGENT (TRI118, Lab Empire) according to the manufacturer’s protocol. The concentration and purity of the isolated RNA were determined via a ND-1000 spectrophotometer (NanoDrop), and the A260/280 and A260/230 ratios were calculated. The quality of the RNA was verified by electrophoresis in a 1% agarose gel, which included SimplySafe staining.

One microgram of total RNA was reverse transcribed using 200 units/ml of M-MLV reverse transcriptase (Sigma‒Aldrich) according to the manufacturer’s protocol, using a GenAmp thermocycler (PerkinElmer, Inc.). Next, cDNA was amplified in qPCR via the Eco Illumina thermocycler (Illumina) with KAPA SYBR^®^ FAST qPCR Master mix (Kapa Biosystems, Inc.) under the following conditions: polymerase activation at 95°C for 10 min, 40 cycles of denaturation at 95°C for 15 sec, and annealing and elongation at 60°C for 30 sec. For normalization of each sample, ribosomal protein S13 (RPS13) cDNA was used. Quantification was performed using the “ΔΔCt” relative quantitation method. All samples were run in triplicate. The experiments were performed three times for quantification of ABCB1 mRNA and four times for quantification of PHLDA1 mRNA. ABCB1, PHLDA1 and RPS13 primers used for qPCR were designed via Primer-BLAST (http://www.ncbi.nlm.nih.gov/tools/primer-blast/)^43^. The sequences of the primers used were as follows: ABCB1 Forward: 5’ GGAAGCCTGAGCTCATTCGAG 3’; Reverse: 5’ TTCAAGATCCATTCCGACCTCGC 3’; PHLDA1 Forward: 5’ CCGGGCAAGACAAGGTTTTGA 3’; Reverse: 5’ CGCAAGTTTTCAGTAGGGTGA 3’; RPS13 Forward: 5’ TGAGAGGAACAGAAAGGATAAGG 3’; Reverse: 5’ ATTTATGCGACCAGGGCAGA 3’. Before the experiments, the efficiency of the reaction for each pair of primers was checked. The cDNA was serially diluted 5-5000, qPCR was performed, and the efficiency of the reaction was calculated in MS Excel via the equation E = 10^(− 1/slope)^. E = 2 specifies 100% efficiency. Only starters for which the calculated reaction efficiency was greater than 95% were used in the experiments.

### ABCB1 activity tests

Doxorubicin is a chemotherapeutic agent that is actively removed from cells by the ABCB1 efflux pump^44^. The cells were treated with a 30 nM solution of doxorubicin (sc-200923, Santa Cruz) (dose selected on the basis of a previous publication^21^ or water (solvent) and then seeded at a density of 1 × 10^6^ cells in 5 ml of medium. The mixture was incubated for 48 h at 37 °C, after which ATP and P21 protein levels were measured as described above. The experiments were performed three times.

The ABCB1 efflux pump actively removes foreign substances from the inside of the cells^44^. One such substance is rhodamine 123, which possesses fluorescent properties. The cells were seeded at a density of 1 × 10^6^ cells in 5 ml of medium and incubated overnight. Next, the cells were treated for 4 h with a 1 µM solution of rhodamine 123 (83702, Sigma‒Aldrich), washed with PBS, and observed via a fluorescence microscope (Nikon Eclipse Ti-S, Japan). The excitation and emission wavelengths were 485 and 539 nm, respectively. The experiments were performed three times.

### Preparation of *PHLDA1*-silenced and control IMR-32 cells for injection into mice

PHLDA1-silenced (shP) and control (shC) cells were grown in 150 cm^2^ culture flasks. The cells were harvested via TrypLeTM reagent (12604021, Gibco) incubation for 1–2 min at 37 °C. Afterwards, the enzyme was inactivated with 10% FBS in EMEM (with 1% NEAA, 1 mM sodium pyruvate) without the antibiotics gentamycin and puromycin, and the cells were subsequently centrifuged (200 × g, 5 min). The pellets were resuspended in PBS. The pools of all resuspended cells were mechanically dispersed to dissociate clumps (as described in^13^) and diluted in PBS for cell counting in a Bürker counting chamber. The excess cells were then rewashed with PBS (200 × g, 5 min). After the removal of the PBS, fresh PBS was added, and the cells were recounted to a specific number for an experiment (with the appropriate excess). Confirmation of PHLDA1 levels in modified cells prepared for injection into mice was performed via western blotting. ShP and shC IMR-32 cells (1 × 10^6^ per well of a 6-well plate) were seeded and cultured for 48 h. Afterwards, the cells were harvested and lysed in RIPA buffer supplemented with protease and phosphatase inhibitors. After PHLDA1 detection, the membrane was stripped, and then α-tubulin was detected (Supplementary Fig. S14, S15 online).

### In vivo experiments and tissue preparation

To generate xenograft tumors, athymic nude mice (Rj: ATHYM-Foxn1nu/nu, Janvier Lab, France) were used on the basis of procedures approved by the 2nd Local Animal Ethical Committee in Kraków, Poland (Approval no. 153/2022). All the experiments were performed in accordance with the relevant guidelines and regulations. The authors complied with the ARRIVE guidelines. The mice were housed under pathogen-free conditions in a temperature-controlled (approximately 22 °C) animal facility with a 14/10 hour light/dark cycle (in ventilated cages, with 55 ± 10% humidity) and were fed *ad libitum*. The mice were allocated to cages (4 animals per cage) by the animal care staff, but no other blinding/randomization was applied by the researchers. The animals were housed in the same rack and at the same height for a given experiment. No criteria for the exclusion or inclusion of animals were set a priori, and no animals were excluded during the described experiments. Each of the 2 separate experiments described here involved 16 mice in 2 groups, which were named after the treatment received. Each experiment included a control group. The mice (8 females per group, approximately 6 weeks old) were subcutaneously (s.c.) injected without anesthesia in the right flank with 4 × 10^6^ shP or shC IMR-32 cells in Matrigel Matrix HC (354262, Corning; the Matrigel was diluted 1:1 with cells in PBS, and the injection volume was 200 µl). The s.c. tumors were monitored at the inoculation site via caliper measurements to ensure that the tumors did not exceed a volume of 1500 mm^3^. The weight was monitored to avoid weight loss of more than 20%. At the endpoint, the animals were euthanized with carbon dioxide (CO_2_), and the tumors were collected, weighed, and processed immediately. The collected tumor samples were incubated for 24 h in 10% formalin (114321730, Chempur), dehydrated in increasing concentrations of ethanol and xylene, and then embedded in paraffin. The paraffin blocks were stored at 4 °C. The paraffin blocks were briefly frozen at -20 °C before being cut on a microtome.

### Histochemical staining

To examine the histology of the tumors, formalin-fixed, paraffin-embedded (FFPE) 4 μm tumor slices were stained with hematoxylin and eosin. To examine the collagen net within the tumors, FFPE slices were stained with a Masson trichrome kit (3459.1, Carl Roth GmbH + Co. KG) and a van Gieson Trichrome kit (8275.1, Carl Roth GmbH + Co. KG) according to the manufacturer’s protocol. Both methods stain connective tissue, but Masson–Goldner staining allows for the visualization and differentiation of muscles and connective tissue (connective tissue is green, muscles are brick red), whereas van Gieson staining differentiates collagen and elastin fibers (elastic fibers are black, and collagen fibers are red).

### Immunohistochemistry

PHLDA1, ABCB1, cleaved PARP-1 and cleaved caspase-3 immunohistochemical reactions were performed via standard formalin-fixed, paraffin-embedded (FFPE) 4 μm slices of neuroblastoma tumors. ABCB1 antigens were retrieved using low-pH buffer (H-3300, Antigen Unmasking Solution, Citrate-based, pH 6.0; Vector Laboratories). Endogenous peroxidase activity was blocked with 3% H_2_O_2_ solution. Membrane permeabilization was achieved with 0.1% Triton X-100 reagent. The tissue sections were incubated overnight with a 1:100 primary antibody mixture (see Table 1) at 4 °C. The next day, the sections were incubated for 30 min with secondary anti-rabbit antibodies (MP-7451, Vector Laboratories). As positive controls, mouse liver and kidney sections were used. PHLDA1, cleaved PARP-1 and cleaved caspase-3 antigens were retrieved using high-pH buffer (H-3301, Antigen Unmasking Solution, Tris-based, pH 9.0; Vector Laboratories). Endogenous peroxidase activity was blocked with 3% H_2_O_2_ solution. The samples were incubated overnight with a 1:40 (PHLDA1), 1:75 (cleaved PARP-1) or 1:50 (cleaved caspase-3) primary antibody mixture (see Table 1). For PHLDA1 and cleaved PARP-1, the sections were incubated overnight at 4 °C, and for cleaved caspase-3, they were incubated for 3 h at room temperature. Then, the sections were incubated for 30 min with secondary anti-rabbit antibodies (MP-7451, Vector Laboratories). For PHLDA1, positive controls for immunostaining of mouse kidney sections were used. The neuroblastoma with the greatest number of morphologically detected apoptotic cells served as a positive control for cleaved PARP-1 and cleaved caspase-3. The ImmPACT Nova Red (SK-4805, Vector Laboratories) reagent was used to visualize the immunostaining reaction. Nuclei were counterstained with Mayer’s hematoxylin. For PHLDA1 and ABCB1 staining the area of the tumors was examined in five random fields of vision for each group and, the both staining intensity, evaluated as 0-negative, 1-weak, 2-moderate, 3-strong. Then the percentage of IHC-positive cells was assessed and used for the staining index calculation according to the formula: [% x staining intensity].

### Assessment of apoptosis

Apoptosis in hematoxylin and eosin-stained samples was identified based on morphology. The number of cells with pyknotic nuclei and apoptotic bodies was counted under a bright-field microscope (Olympus Optical Co., Tokyo, Japan) with a 40x objective. Five adjacent fields of view present on each slide were examined. Apoptotic cells were also detected with an anti-cleaved PARP-1 antibody and an anti-cleaved caspase-3 antibody, and the number of cells with a positive IHC signal was counted with a 40x objective. The results of the counting of apoptotic cells are shown in the Supplementary Table S2a-f online.

### Bioinformatic analyses

Bioinformatic screening was performed via the R2 platform (http://r2.amc.nl, http://r2platform.com). The Versteeg 88 dataset was used for the analyses^30^.

### Statistical evaluation

Each of the in vitro experiments was conducted at least three times. The results are presented as the means (± SEM) of independent experiments. Statistical analyses of experiments involving two groups were performed via two-tailed Student’s t test for independent samples. Other experiments were statistically analyzed via the Kruskal‒Wallis ANOVA test, followed by Dunn’s post hoc analysis or one-way ANOVA followed by the post hoc Tukey’s test. Calculations were performed via Excel software (Microsoft), R (R version 3.2.1 Patched), and Graph Pad Prism 6.

## Supplementary Information

Below is the link to the electronic supplementary material.

Supplementary Material 1

## Data Availability

The data are provided within the manuscript and in the Supplementary Information online. Complete data generated in the present study may be requested from the first or corresponding author.
