# Supplementary material for: PHLDA1 silencing in IMR-32 human neuroblastoma cells results in ABCB1 overexpression, augments chemoresistance and leads to increased growth of tumors
Source: Sci Rep. 2025 Dec 22;15:44427. doi: 10.1038/s41598-025-33551-0 (PMC12738729; doi:10.1038/s41598-025-33551-0)
Supplement: Supplementary file 1 — Supplementary Material 1 [file 41598_2025_33551_MOESM1_ESM.pdf]

Supplementary Information

Kudrycka, M.; Horwacik, I.; Brożyna, A.A.; Żywicka, M.; Durbas, M.; Rokita, H.

***PHLDA1* silencing in IMR-32 human neuroblastoma cells results in *ABCB1* overexpression, augments chemoresistance and leads to increased growth of tumors**

Fig. S1. The expression level of *ABCB1* and *PHLDA1* in *PHLDA1*-silenced IMR-32 cells

3 repetitions of qRT-PCR for the *ABCB1* level

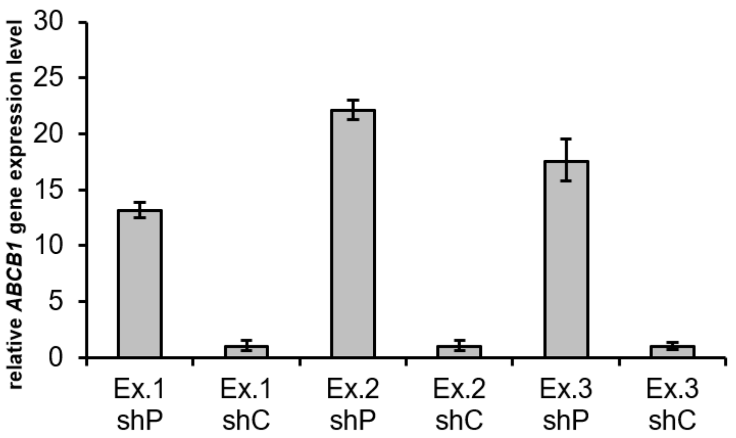

4 repetitions of qRT-PCR for the *PHLDA1* level

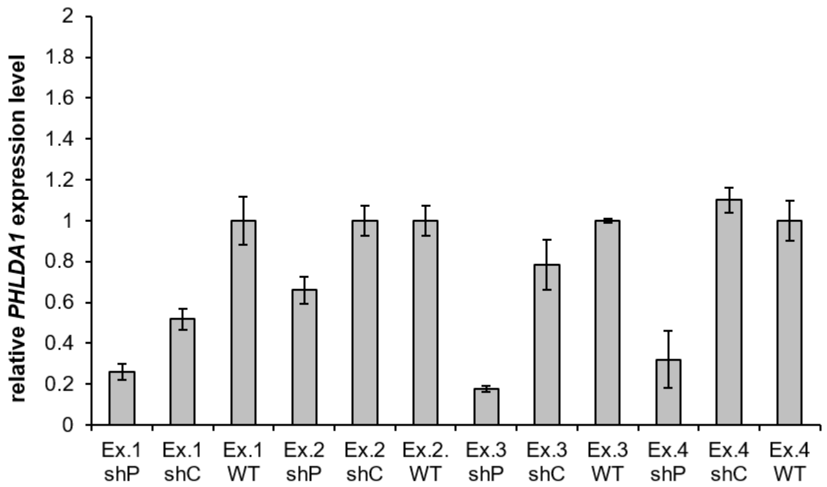

ABCB1 and *PHLDA1* gene expression analysis in *PHLDA1*-silenced IMR-32 cells (shP), control cells (shC) and wild-type cells (WT). The *ABCB1* mRNA content in shC was set as 1. The *PHLDA1* mRNA content in the WT was set as 1. The samples were run in triplicate. The data are shown as the means (±SEM) of 3 independent experiments (for the quantification of the *ABCB1* mRNA) and 4 independent experiments (for the quantification of the *PHLDA1* mRNA).

Fig. S2. The level of ABCB1 and PHLDA1 in *PHLDA1*-silenced IMR-32 cells

Complete data – 3 repetitions with HepG2 as a positive control for ABCB1

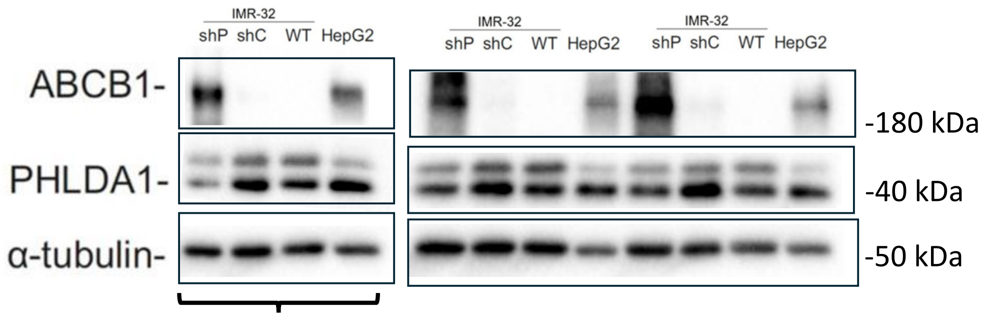

The representative immunoblots presented in Fig.1c

Raw western blots for Fig. S2.

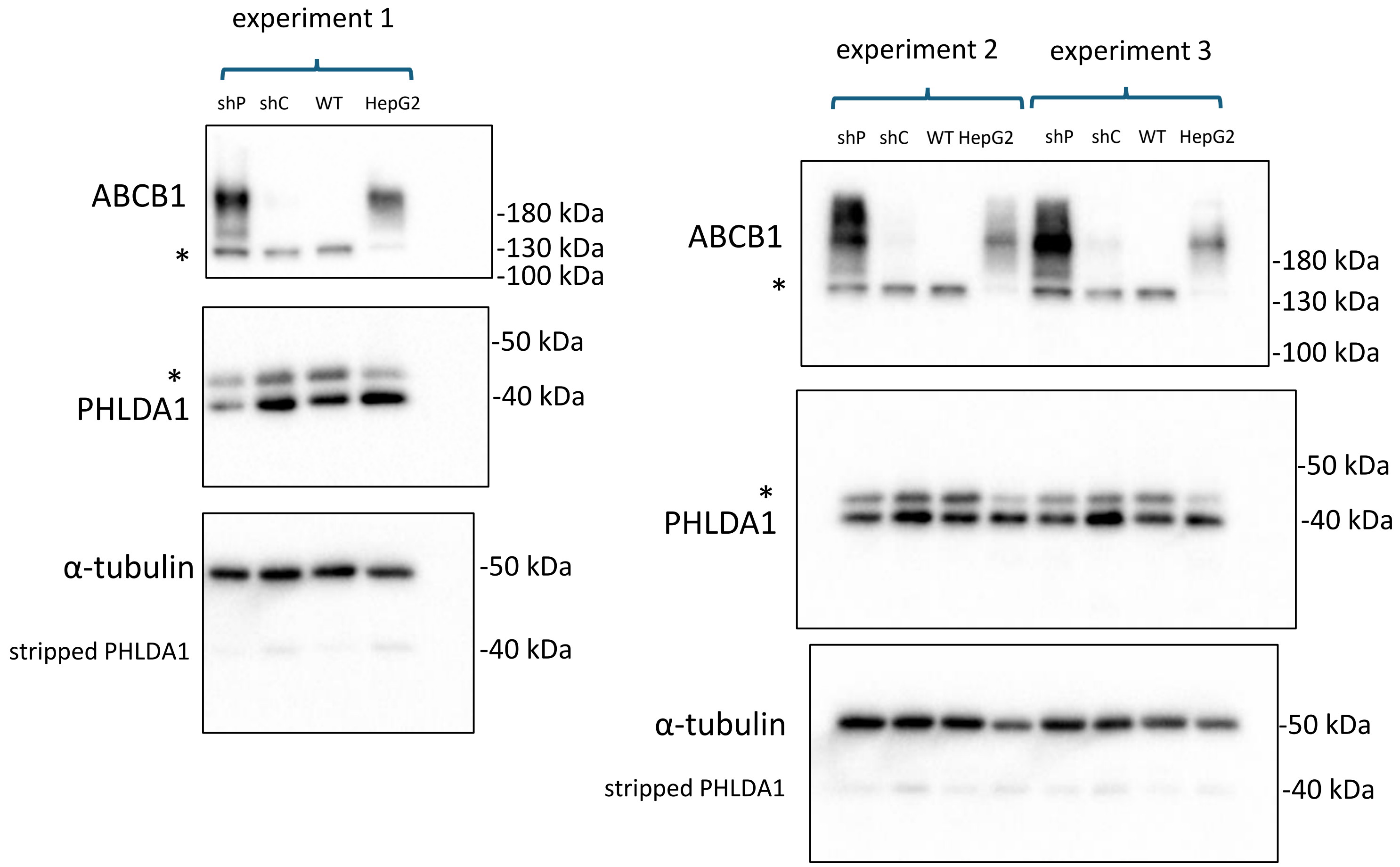

Western blot analysis of ABCB1 and PHLDA1 proteins in shP, shC and WT IMR-32 cells. The HepG2 cell line was used as a positive control for the ABCB1 protein. α-Tubulin was used as a reference. Immunoblots of 3 independent experiments are shown. The cells were cultured for 48 hours, harvested and lysed with RIPA buffer. 20 µg of protein lysate was used per well. The membranes were cut before incubation with antibodies. Abbreviations: shP – cells with *PHLDA1*-silencing, shC – control cells, WT – nontransfected cells, HepG2 – HepG2 cells, * - unspecific band.

Fig. S3. Raw immunoblots for Fig. S2

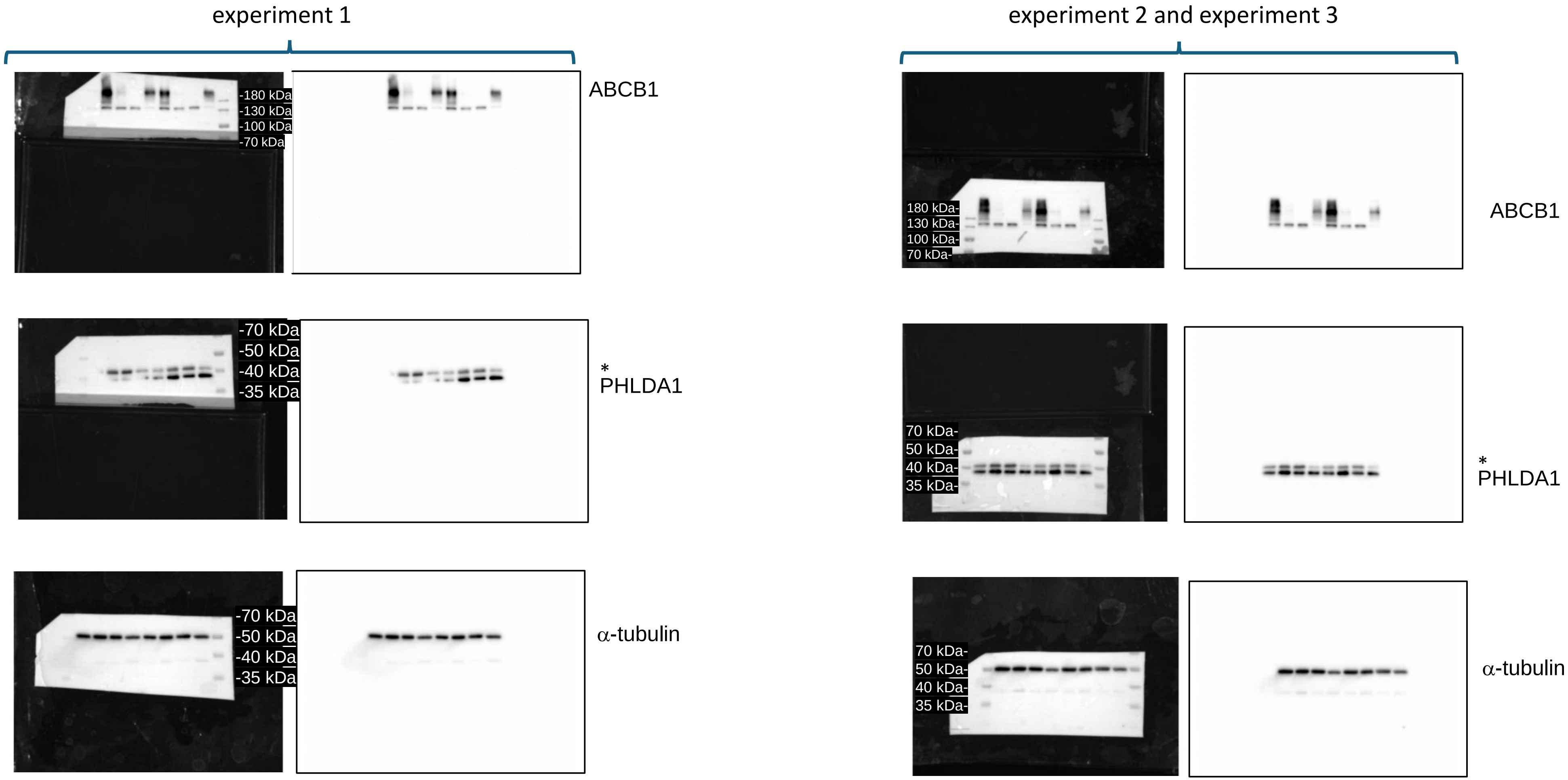

The original blots for Fig. S2 are shown. Membranes were cut prior to incubation with antibodies detecting ABCB1, PHLDA1 and α-tubulin, respectively. For each experiment the photographs of the cut membranes (with visible edges of the blots and molecular weight marker bands) collected in the visible light were merged with the photographs of the chemiluminescent signals (see the left panels). The chemiluminescent signals alone are shown in the right panels. * - unspecific band.

Fig. S4. Verification of specificity of two anti-PHLDA1 antibodies (Sc-23866 from Santa Cruz Biotechnology and ab133654 from Abcam) used in the study

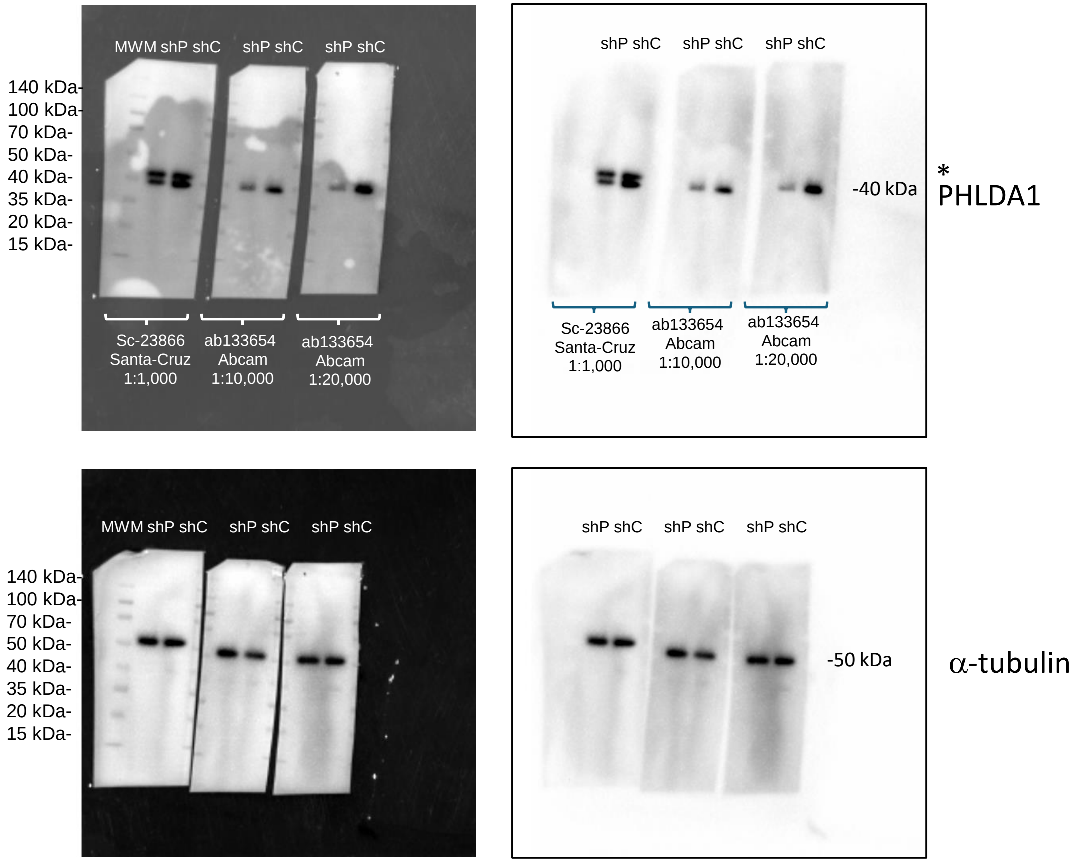

Western blot analysis of PHLDA1 proteins in shP and shC IMR-32 cells to validate specificity of anti-PHLDA1 antibodies (Sc-23866 from Santa Cruz Biotechnology and ab133654 from Abcam). The membrane was cut before incubation with antibodies. α-Tubulin was used as a reference protein. 20 µg of protein lysate was used per well. The photographs of the cut membranes (with visible edges of the blots and molecular weight marker bands) collected in the visible light were merged with the photographs of the chemiluminescent signals (see the left panel). The chemiluminescent signals alone are shown in the right panel. Abbreviations: MWM – molecular weight marker, shP –*PHLDA1*-silenced cells, shC – control cells, * - unspecific band.

Fig. S5. The level of ABCB1 and PHLDA1 in another *PHLDA1*-silenced neuroblastoma cell line - CHP-134

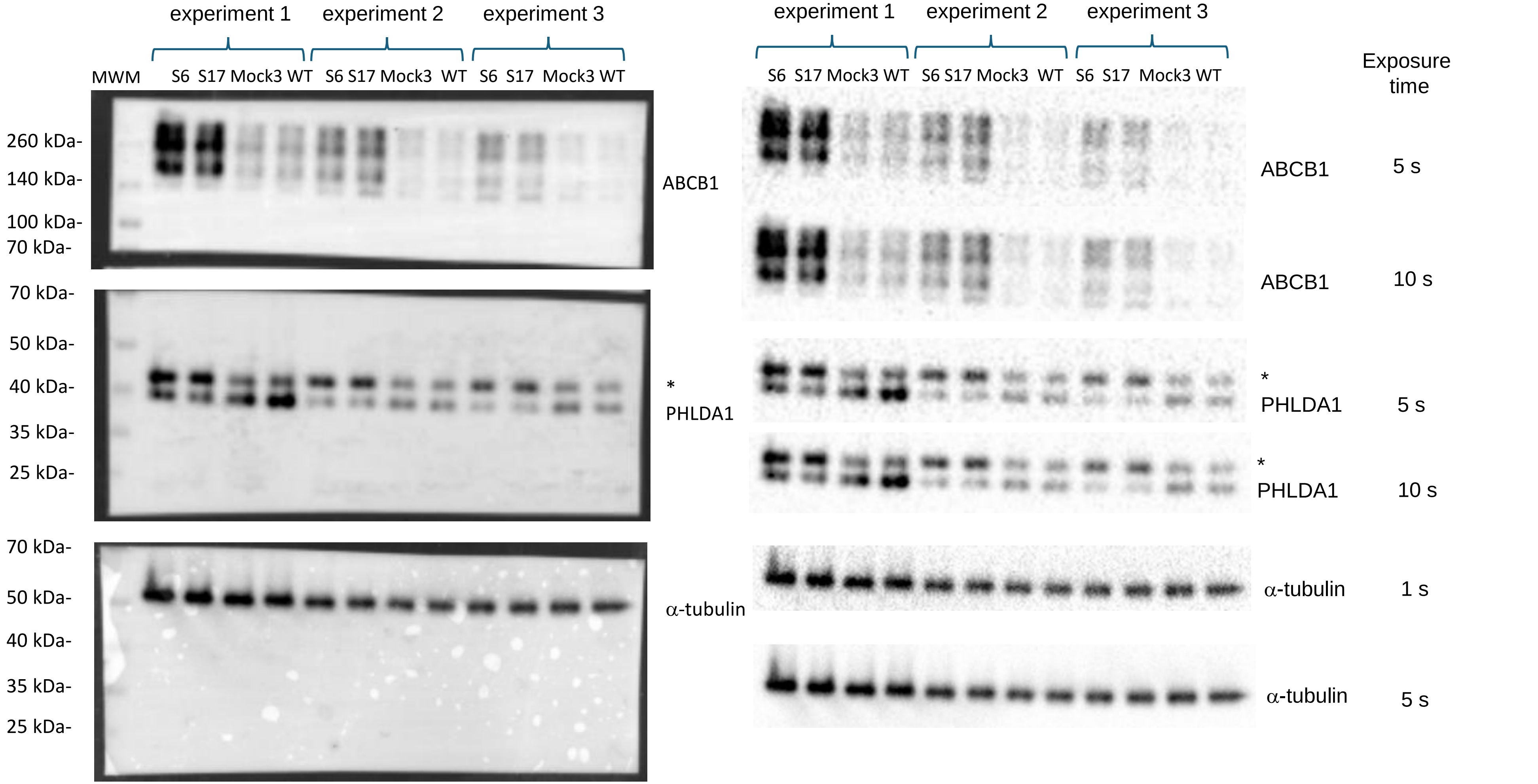

Western blot analysis of ABCB1 and PHLDA1 proteins in *PHLDA1*-silenced (the S6 and S17 clones), control (the Mock3 clone) and WT (non-transduced) CHP-134 cells. α-Tubulin was used as a reference. Cells were cultured for 48 hours, harvested and lysed with RIPA buffer. 5 µg of protein lysate was used per well. The original blot was cut prior to incubation with antibodies. The photographs of the cut membranes (with visible edges of the blots and molecular weight marker bands) collected in the visible light were merged with the photographs of the chemiluminescent signals (see the left panel). The chemiluminescent signals alone are shown in the right panel. Immunoblots of 3 independent experiments are shown with 2 exposure times for each protein detected. Abbreviations: MWM – molecular weight marker, * - unspecific band.

Fig. S6. The rhodamine 123 assay for ABCB1 efflux pump activity in *PHLDA1-silenced* IMR-32 cells

3 repetitions of the rhodamine 123 assay - microphotographs

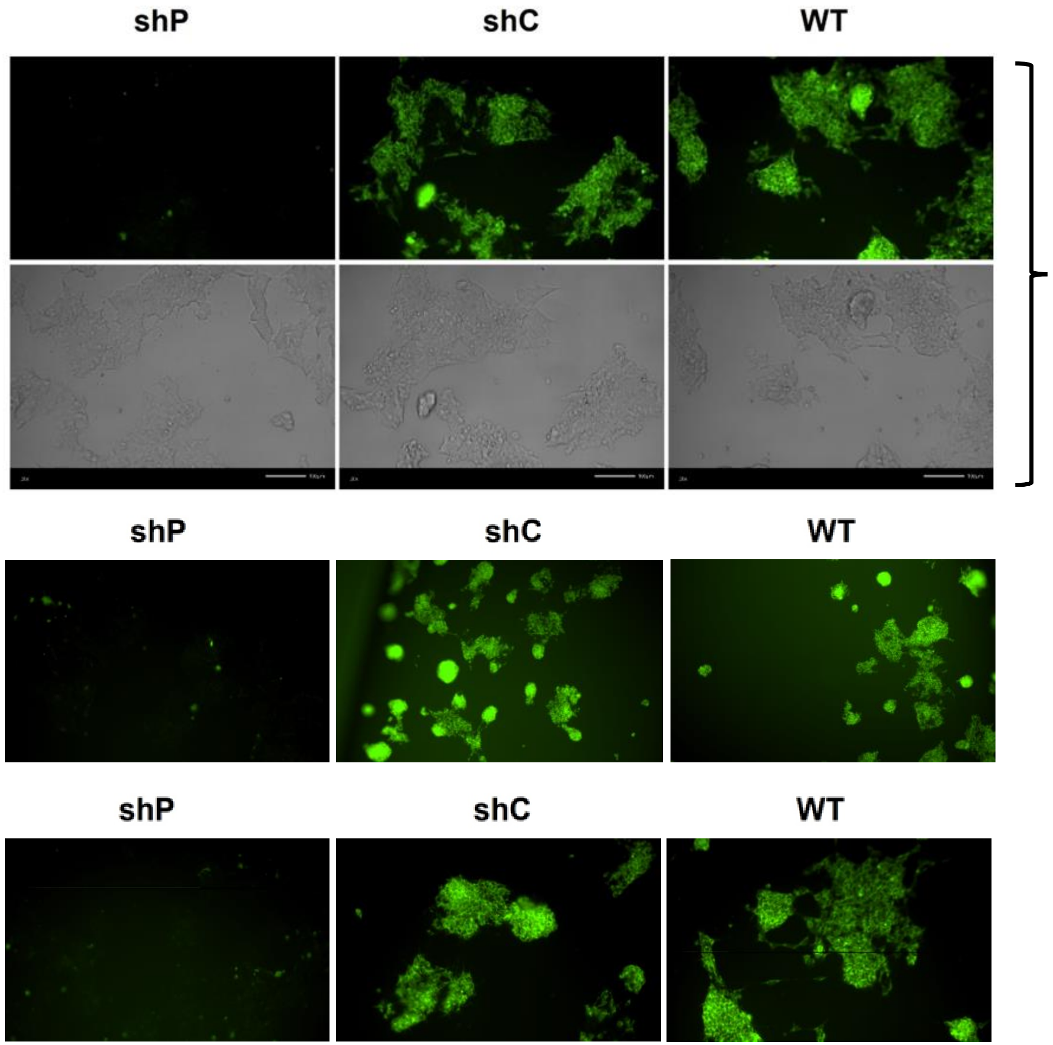

The microphotographs presented in Fig. 2a.
The brightfield microphotographs were added
for the representative images with fluorescent signal.

ABCB1 activity in IMR-32 cells with *PHLDA1*-silencing. ABCB1 activity was verified via a rhodamine 123 accumulation assay and visualized via fluorescence microscopy. The corresponding cell images of *PHLDA1*-silenced (shP), control (shC), and WT IMR-32 cells are shown in the fluorescence mode for all 3 experiments. Brightfield microphotographs were taken during one experiment. Scale bars – 100 µm.

Fig. S7. The doxorubicin assay for ABCB1 efflux pump activity in *PHLDA1-silenced* IMR-32 cells

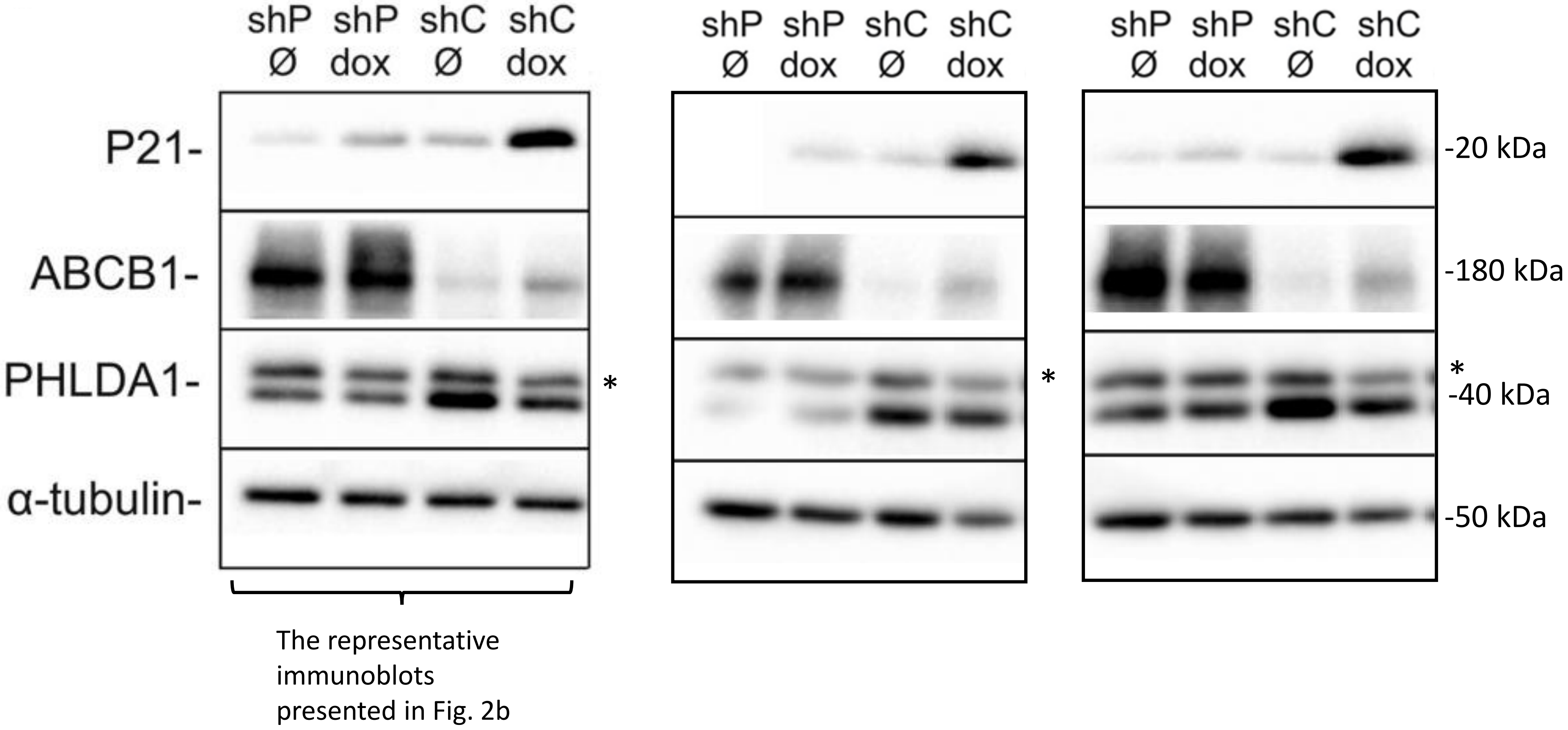

Western blot analysis of P21, ABCB1 and PHLDA1 proteins in shP and shC IMR-32 cells after the ABCB1 functional test of doxorubicin treatment. 3 repetitions of the doxorubicin assay were performed. *PHLDA1*-silenced and control IMR-32 cells were treated with 30 nM doxorubicin or water for 48 hours, protein lysates were subsequently obtained, and analyzed by western blot. The membranes were cut before incubation with antibodies. α-Tubulin was used as a reference protein. Abbreviations: shP – *PHLDA1*-silenced IMR-32 cells, shC – control IMR-32 cells, Ø – cells treated with water (solvent for doxorubicin), dox – cells treated with doxorubicin, * - unspecific band.

Fig. S8. Immunoblots for Fig. S7

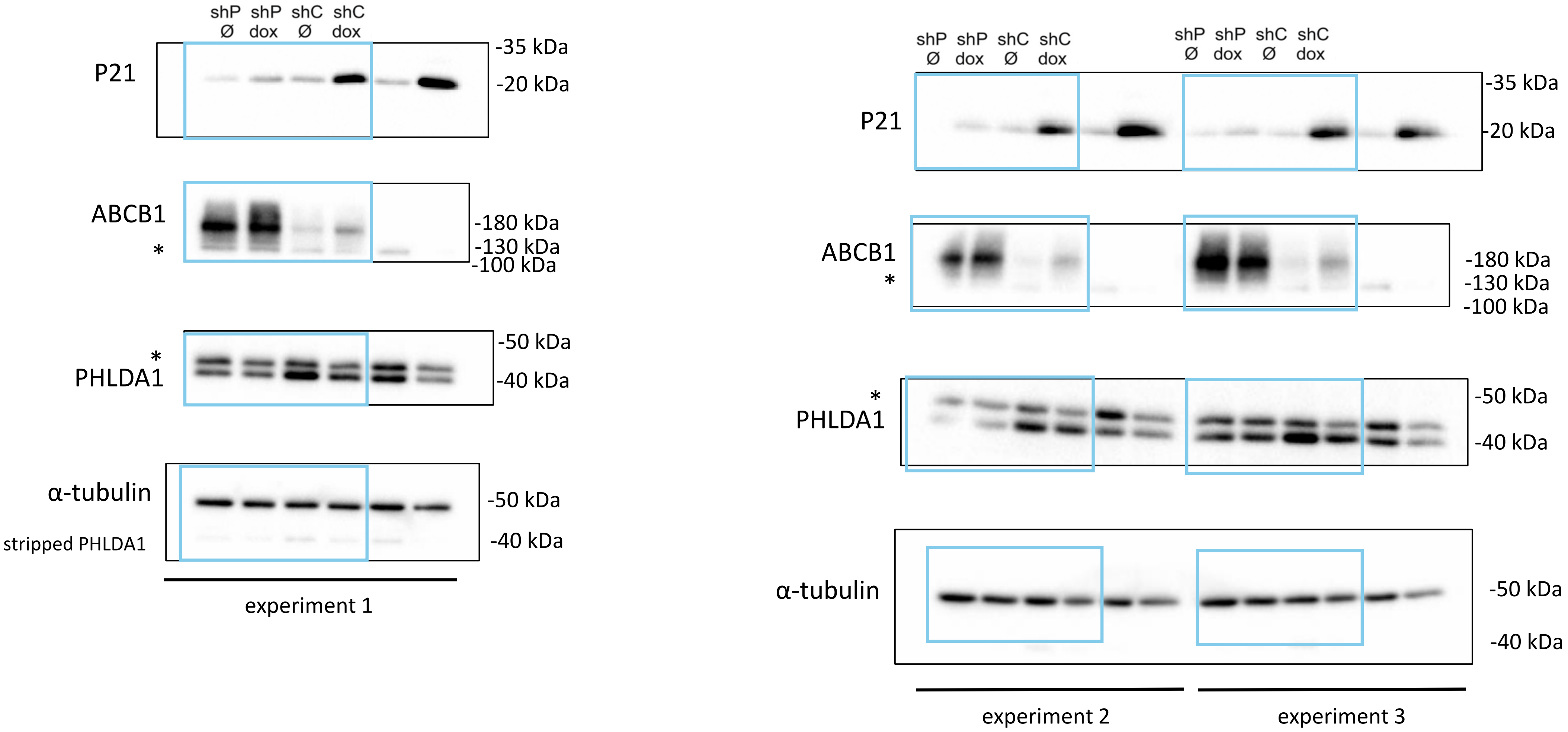

The original blots for Fig. 2b are shown. Membranes were cut prior to incubation with antibodies detecting ABCB1, PHLDA1 and α-tubulin, respectively. The chemiluminescent signals alone are shown. The blue frames depict data presented in Fig. 2b and Fig. S7. Abbreviations: shP – *PHLDA1*-silenced IMR-32 cells, shC – control IMR-32 cells, Ø – cells treated with water (solvent for doxorubicin), dox – cells treated with doxorubicin, * - unspecific band.

Fig. S9. Raw immunoblots for Fig. S7

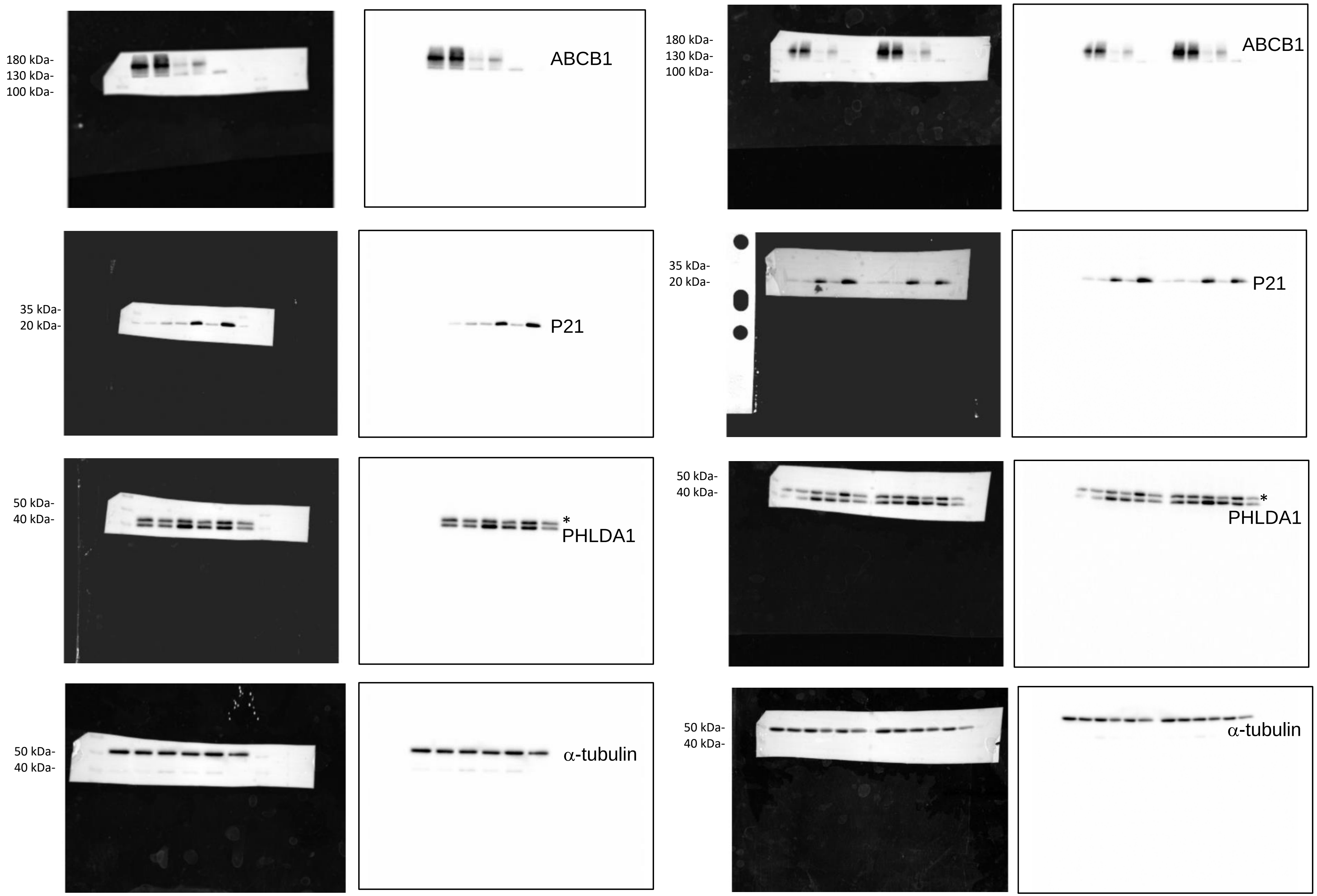

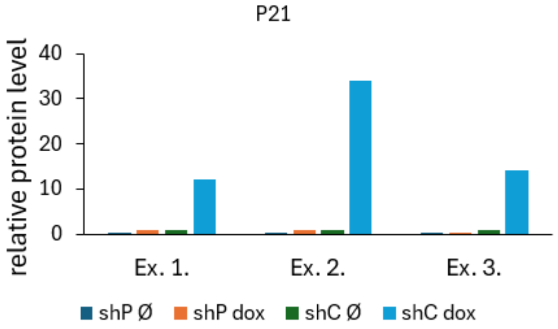

Mean values ±SD for
shP Ø: 0.155 ±0.151
shP dox: 0.644 ±0.371
shC Ø: 1 ±0
shC dox: 20.037 ±12.061

The original blots for Fig. S7 are shown. Membranes were cut prior to incubation with antibodies detecting ABCB1, P21, PHLDA1, and α-tubulin, respectively. For each experiment the photographs of the cut membranes (with visible edges of the blots and molecular weight marker bands) collected in the visible light were merged with the photographs of the chemiluminescent signals (see the left panels). The chemiluminescent signals alone are shown in the right panels. * - unspecific band. Densitometric analyses were performed using the ImageJ software, then calculated in MS Excel (see the plot, mean values with standard deviations ±SD above). Results shown as relative values for shC and normalised to α-tubulin. Abbreviations: shP – *PHLDA1*-silenced IMR-32 cells, shC – control IMR-32 cells, Ø – cells treated with water (solvent for doxorubicin), dox – cells treated with doxorubicin.

Fig. S10. The doxorubicin assay for ABCB1 efflux pump activity in *PHLDA1-silenced* cells

Complete data from 3 experiments presented in Fig. 2c

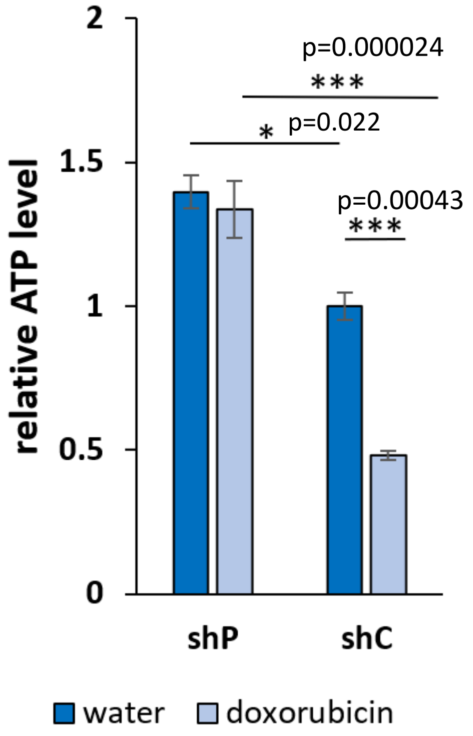

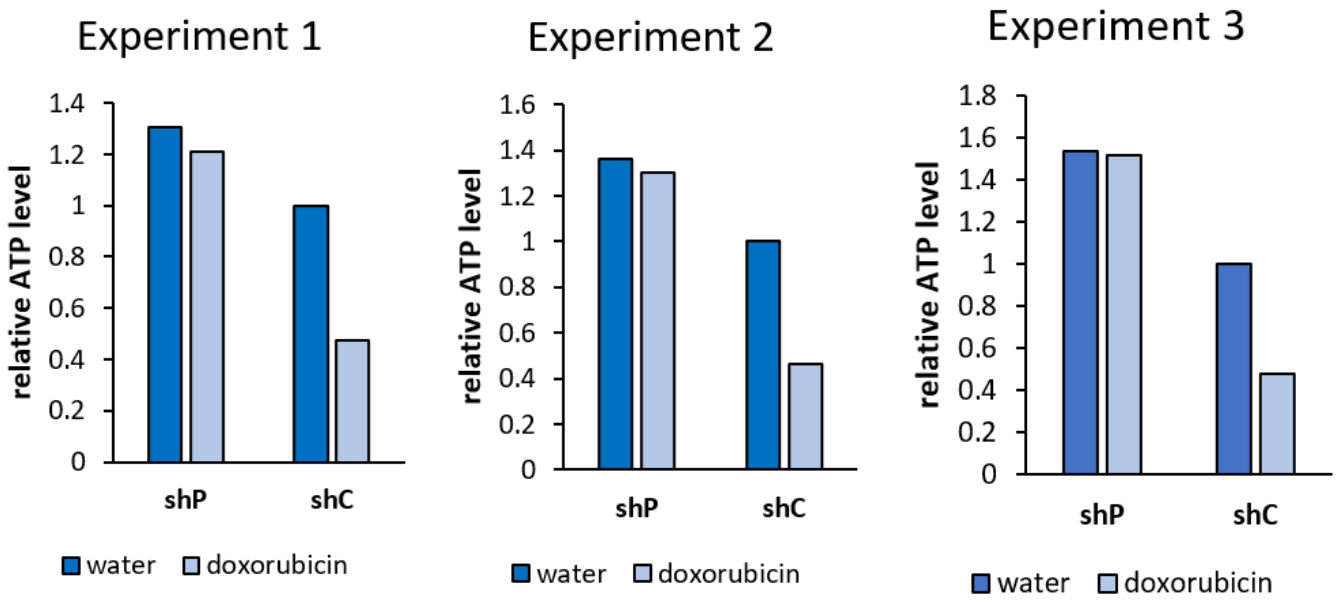

The ABCB1 functional test after doxorubicin treatment. 3 repetitions of the doxorubicin assay were performed. *PHLDA1*-silenced (shP) and control (shC) IMR-32 cells were treated with 30 nM doxorubicin (dox) or water (Ø) for 48 hours and measurements of ATP levels were performed to confirm the cytotoxic effect of doxorubicin. The ATP level in control cells (treated with water) was set to 1. The samples were run in triplicate and mean values are presented for the plots for experiments 1-3. The complete data from the 3 independent experiments are shown as the means (±SEM) with statistical significance assessed with one-way ANOVA, followed by *post hoc* Tukey's test (*p<0.05, ***p<0.001, the exact p values were shown on the plot).

Fig. S11. The response of *PHLDA1*-silenced, control and WT CHP-134 cells to doxorubicin

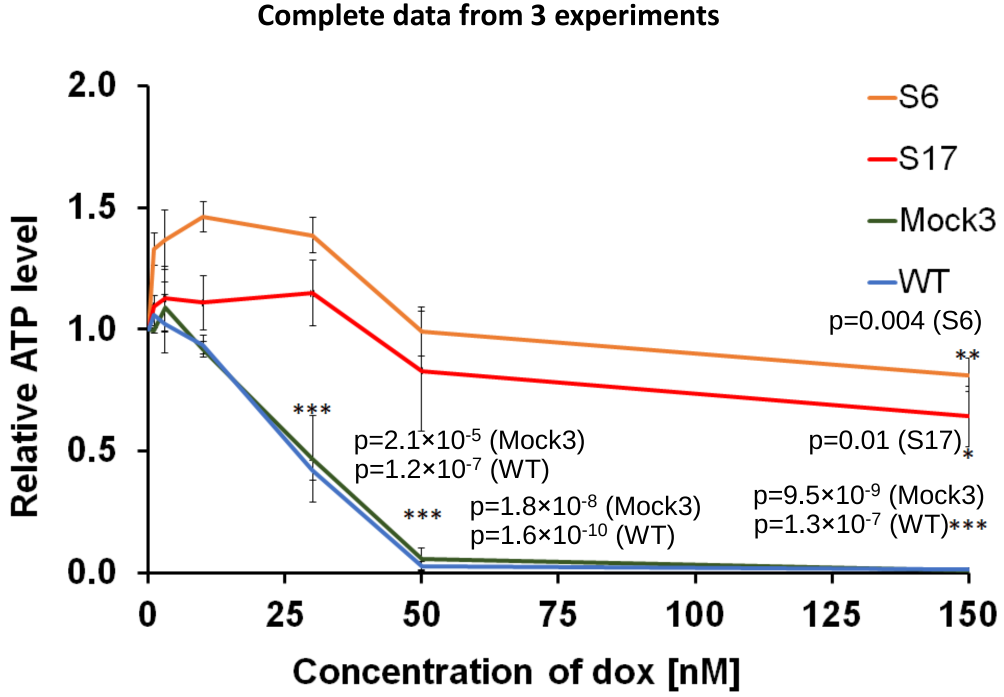

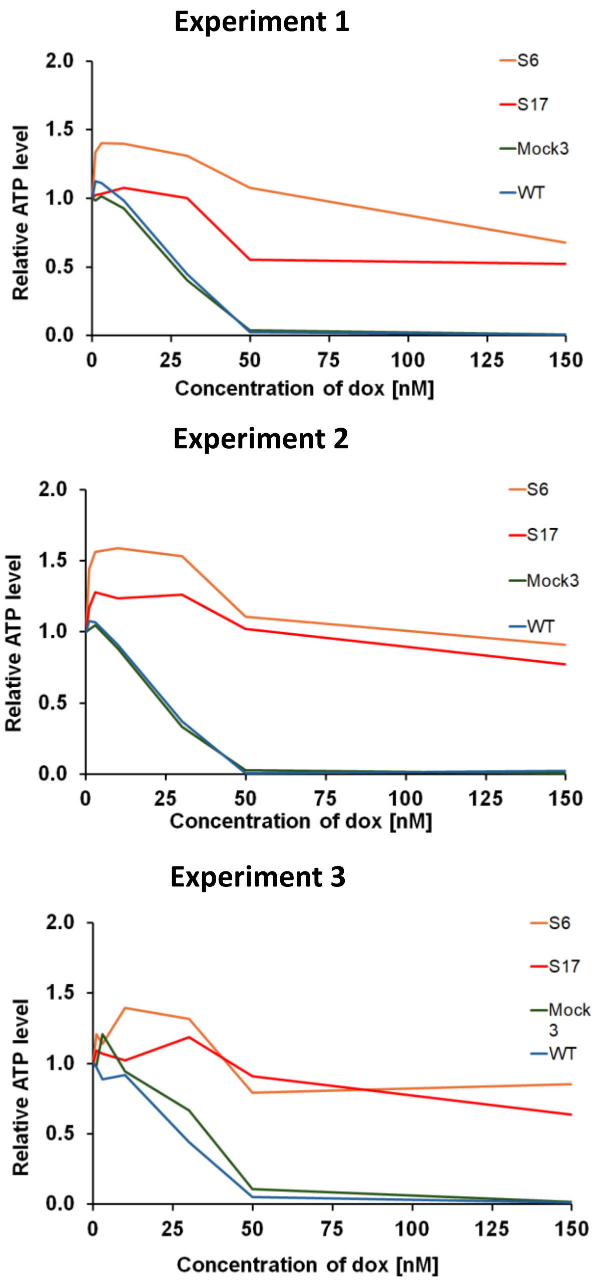

The response of *PHLDA1*-silenced, control and WT (non-transduced) CHP-134 cells to doxorubicin. *PHLDA1*-silenced (the S6 and S17 clones), control (the Mock3 clone) and WT cells were cultured for 48 hours with doxorubicin in the concentration range of 1-150 nM. 3 independent experiments were performed. The samples were run in triplicate and mean values are presented for the plots for experiments 1-3. An ATP luminescence assay was performed to confirm the cytotoxic effect of doxorubicin. ATP content was measured and compared with the respective controls (treated with water, set as 1). The complete data from the 3 independent experiments are shown as the means (±SEM) and statistical significance was calculated with Kruskal-Wallis ANOVA followed by *post hoc* Dunn's test (*p<0.05, **p<0.01, ***p<0.001, the exact p values were shown on the plot). Abbreviations: dox - doxorubicin.

Fig. S12. The protein patterns in *PHLDA1*-overexpressed and control IMR-32 cells treated with to doxorubicin

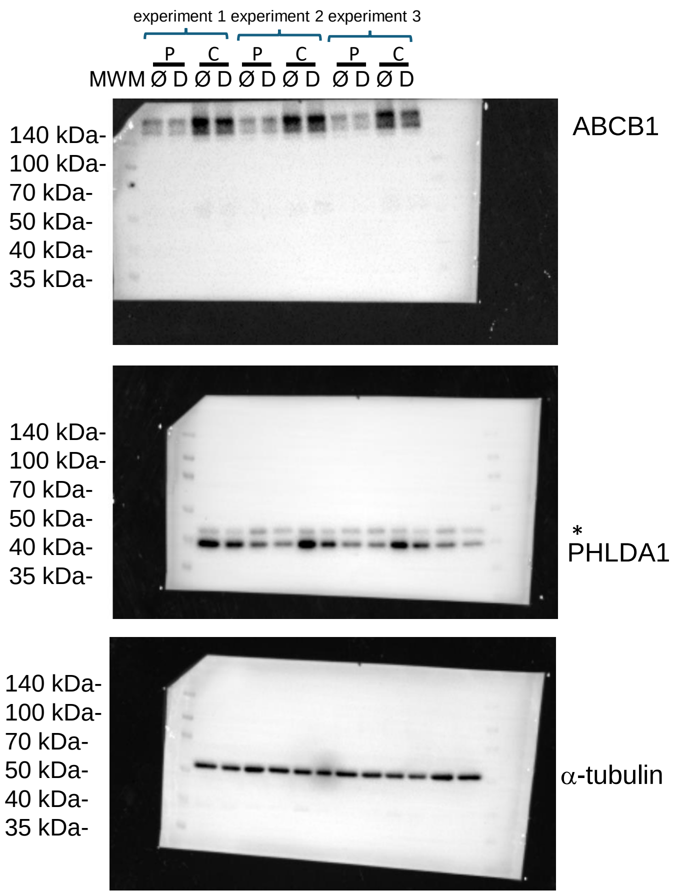

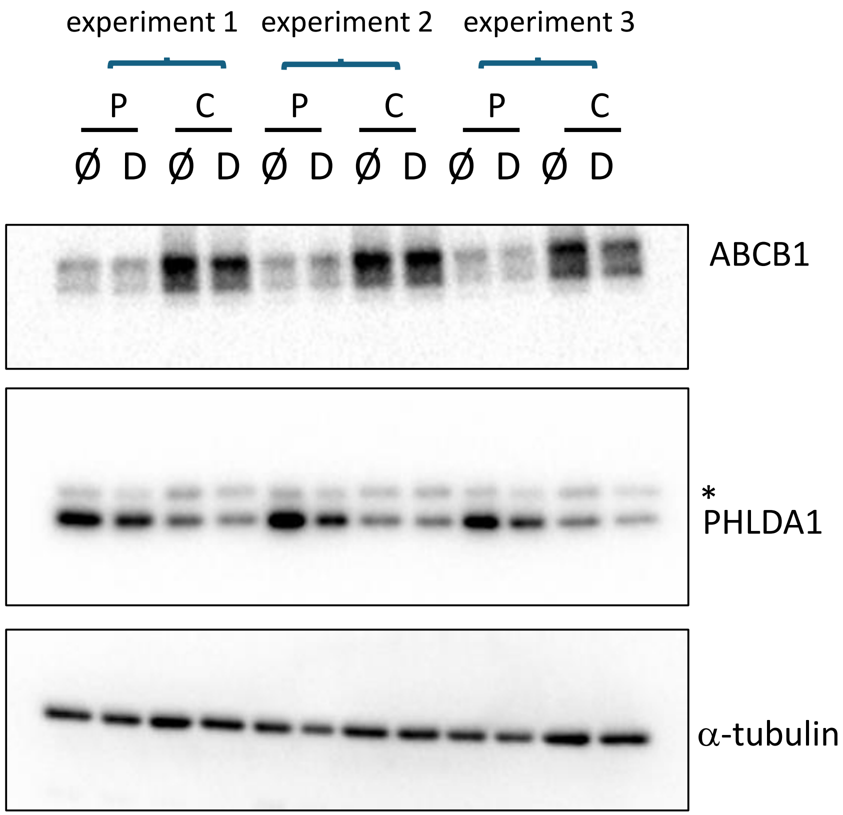

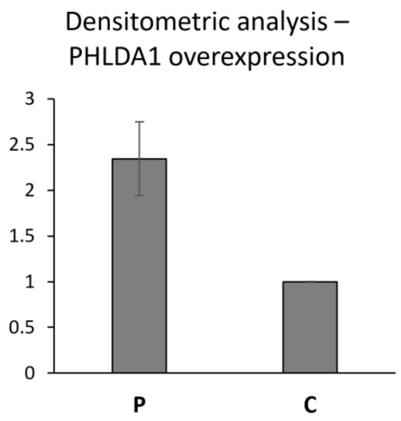

Western blot analysis of PHLDA1 and ABCB1 in IMR-32 cells with stable overexpression of PHLDA1 and control cells treated with 30 nM of doxorubicin or solvent (water) for 48 hours. The cells were harvested and lysed with RIPA buffer. α-Tubulin was used as a reference protein. 20 µg of protein lysate was used per well. Representative photographs of the cut membranes (with visible edges of the blots and molecular weight marker bands) collected in the visible light were merged with the photographs of the chemiluminescent signals (see the left panel). The chemiluminescent signals alone are shown in the right panel. Densitometric analysis of PHLDA1 overexpression, normalized to α-tubulin, was shown on the plot as the means (± SEM) of 3 experiments. The level of PHLDA1 in control cells was set as 1. Abbreviations: MWM – molecular weight marker, * - unspecific band, Ø – water treated cells, D – doxorubicin treated cells, P – IMR-32 cells with stable overexpression of *PHLDA1*, C - control cells.

Fig. S13. The response of *PHLDA1*-overexpressed and control IMR-32 cells to doxorubicin

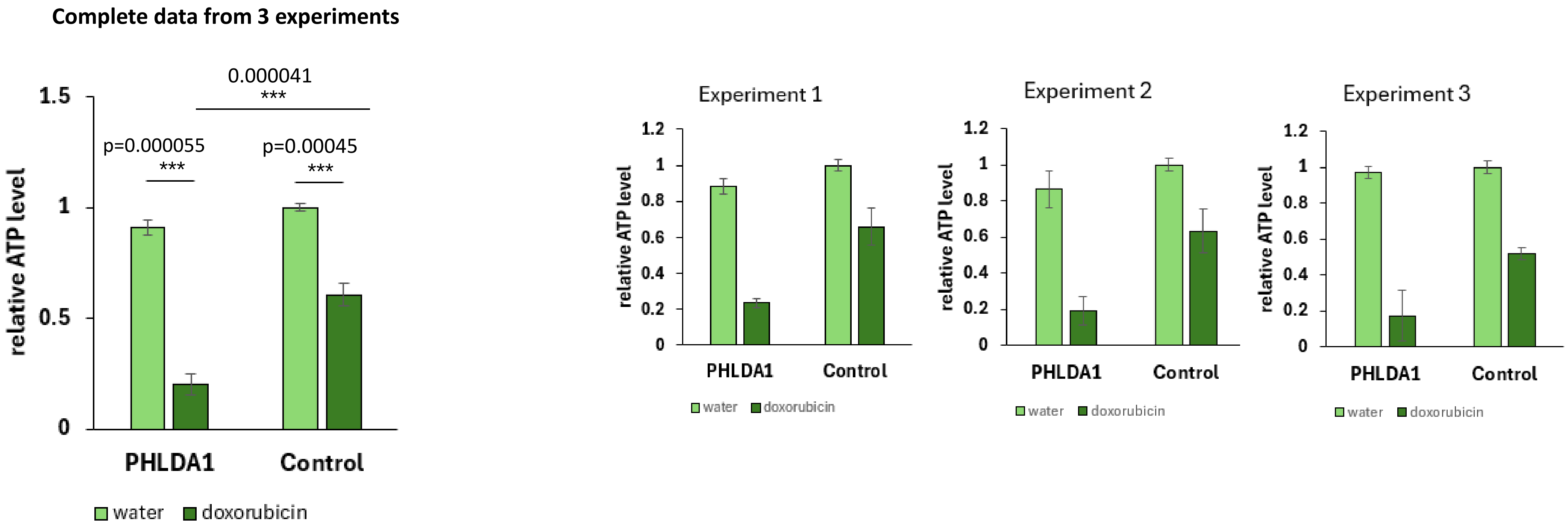

PHLDA1 overexpression causes higher susceptibility to doxorubicin in IMR-32 cells. IMR-32 cells with stable overexpression of *PHLDA1* (PHLDA1 – obtained by transfection with an ORF PHLDA1 plasmid) and control cells (Control – obtained by transfection with an empty plasmid) were treated with 30 nM of doxorubicin or solvent (water) for 48 hours. Then, the ATP level in cells was measured. The ATP level of control cells (treated with water) was set as 1. 3 experiments were performed. The samples were run in triplicate and mean values (±SEM) are presented for the plots for experiments 1-3. The complete data from the 3 independent experiments are presented as the means (±SEM) and statistical significance was measured by Kruskal-Wallis ANOVA with *post hoc* Dunn's test (***$p<0.001$, the exact p values were shown on the plot).

Fig. S14. *PHLDA1*-silencing in IMR-32 cells injected into mice – experiment 1

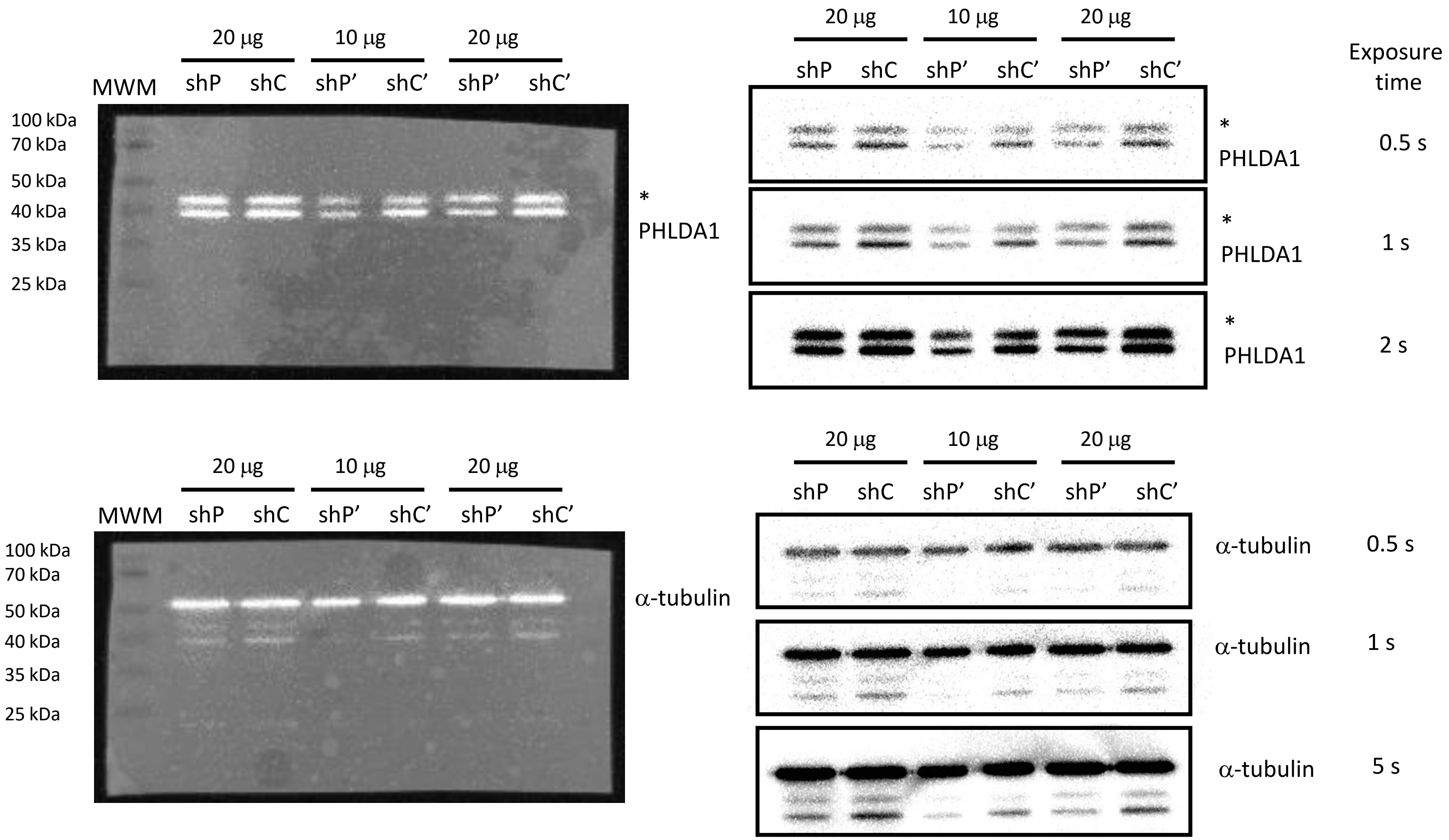

Western blot analysis of PHLDA1 in shP and shC IMR-32 cells – experiment 1. The cells were cultured for 48 hours, harvested and lysed with RIPA buffer. 20 µg of protein lysate was used per well. The original blot was cut prior to incubation with antibodies. The top part (above 100 kDa band of molecular weight marker) and the bottom part of the membrane (below 25 kDa) were cut out to accommodate smaller size containers to limit the volume of antibody. Firstly, PHLDA1 was detected on the membrane. Then the membrane was stripped, and α-tubulin was detected as a reference protein. 10 µg and 20 µg of protein lysate was used per well shP', shC' for optimization. The photographs of the cut membranes (with visible edges of the blots and molecular weight marker bands) collected in the visible light were merged with the photographs of the chemiluminescent signals (see the left panel). The chemiluminescent signals alone are shown in the right panel. Immunoblots are shown with 3 exposure times for each protein detected. Abbreviations: shP – *PHLDA1*-silenced cells; shC – control cells; shP' – *PHLDA1*-silenced cells for optimization, shC' - control cells for optimization, MWM – molecular weight marker, * - unspecific band.

Fig. S15. *PHLDA1*-silencing in IMR-32 cells injected into mice – experiment 2

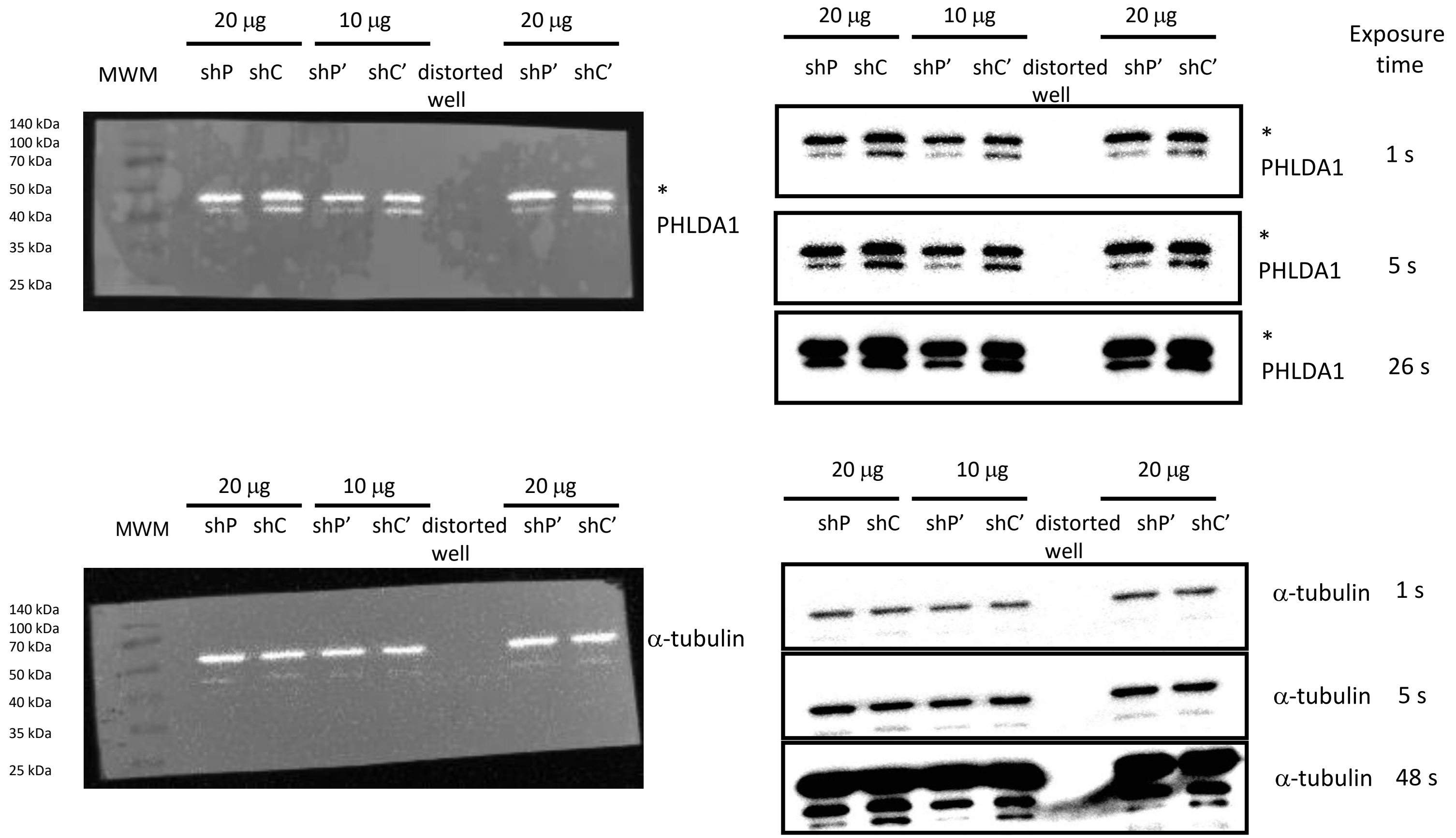

Western blot analysis of PHLDA1 in shP and shC IMR-32 cells – experiment 2. The cells were cultured for 48 hours, harvested and lysed with RIPA buffer. 20 µg of protein lysate was used per well. The original blot was cut prior to incubation with antibodies. The top part (above 140 kDa band of molecular weight marker) and the bottom part of the membrane (below 25 kDa) were cut out to accommodate smaller size containers to limit the volume of antibody. Firstly, PHLDA1 was detected on the membrane. Then the membrane was stripped, and α-tubulin was detected as a reference protein. 10 µg and 20 µg of protein lysate was used per well shP’, shC’ for optimization. The photographs of the cut membranes (with visible edges of the blots and molecular weight marker bands) collected in the visible light were merged with the photographs of the chemiluminescent signals (see the left panel). The chemiluminescent signals alone are shown in the right panel. Immunoblots are shown with 3 exposure times for each protein detected. Abbreviations: shP – *PHLDA1*-silenced cells; shC – control cells; shP’ – *PHLDA1*-silenced cells for optimization, shC’ - control cells for optimization, MWM – molecular weight marker, * - unspecific band.

Fig. S16. Pictures of tumors from the experiments 1 and 2 after their removal from mice

Experiment 1

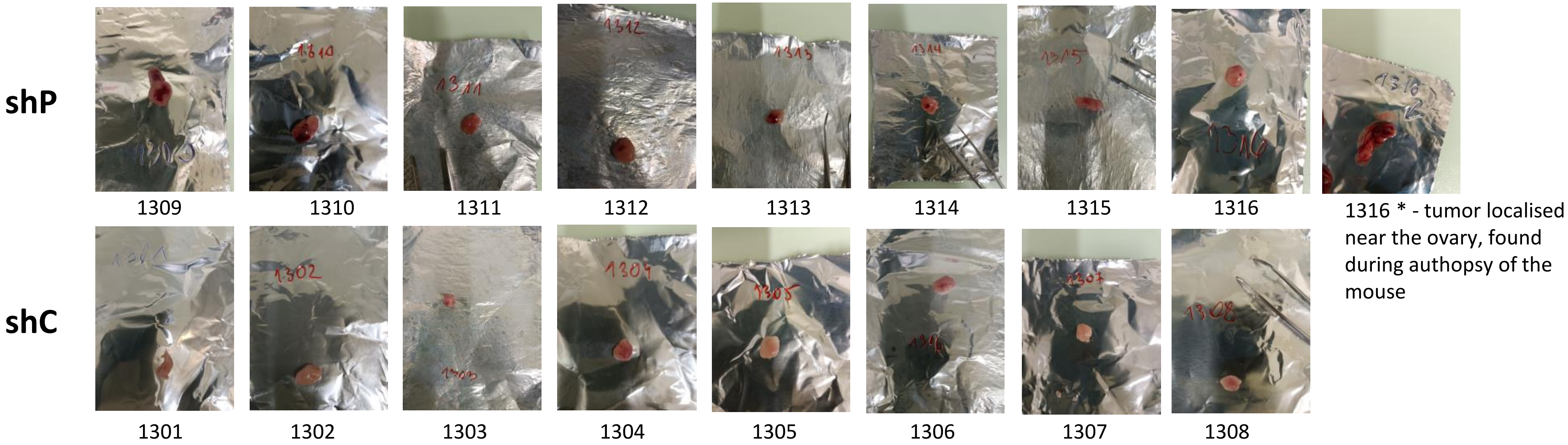

1316 * - tumor localised near the ovary, found during authopsy of the mouse

Experiment 2

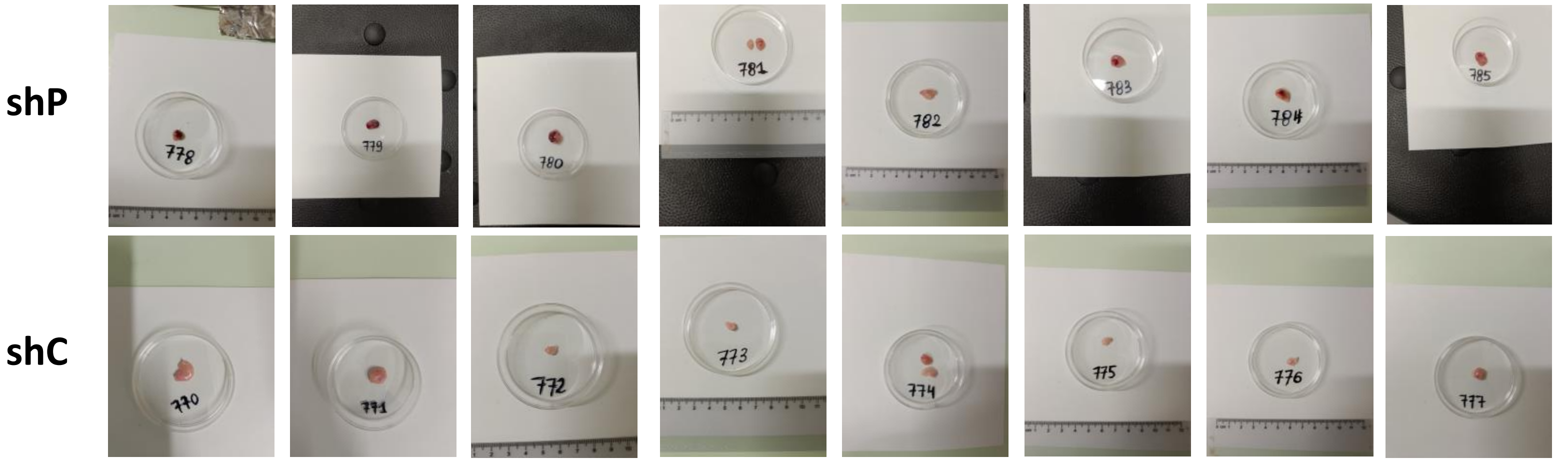

Photographic images of all the tumors harvested during experiment 1 and 2. Mouse numbers were written below photographs or on Petri dishes. The proportions between the tumors cannot be accurately compared due to differences in camera positioning. Abbreviations: shP – tumors derived from *PHLDA1*-silenced IMR-32 cells, shC – tumors derived from control cells.

Fig. S17. Hematoxylin and eosin staining in shP and shC tumors - additional microphotographs than presented in Fig. 3c

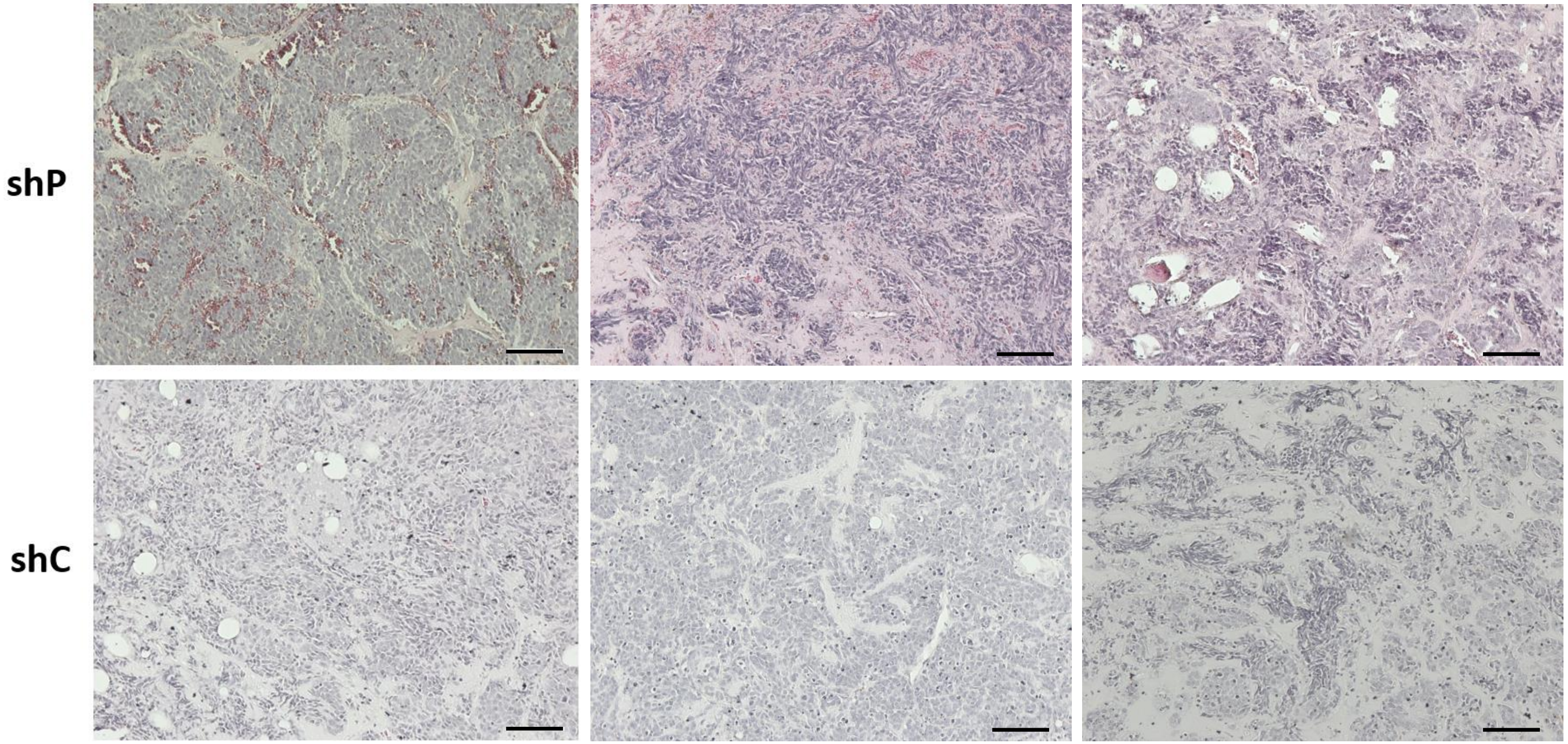

Mouse 1310, 1313, 1316, respectively

Mouse 1303, 1302, 1305, respectively

Microscopic images of slices of xenograft neuroblastoma tumors grown from *PHLDA1*-silenced (shP) and control (shC) IMR-32 cells stained with hematoxylin and eosin (HE) to depict extravasations within shP tumors. Six different tumors were presented with the HE staining. Scale bars – 100 µm.

Fig. S18. Collagen staining in shP and shC tumors - additional microphotographs than presented in Fig. 4

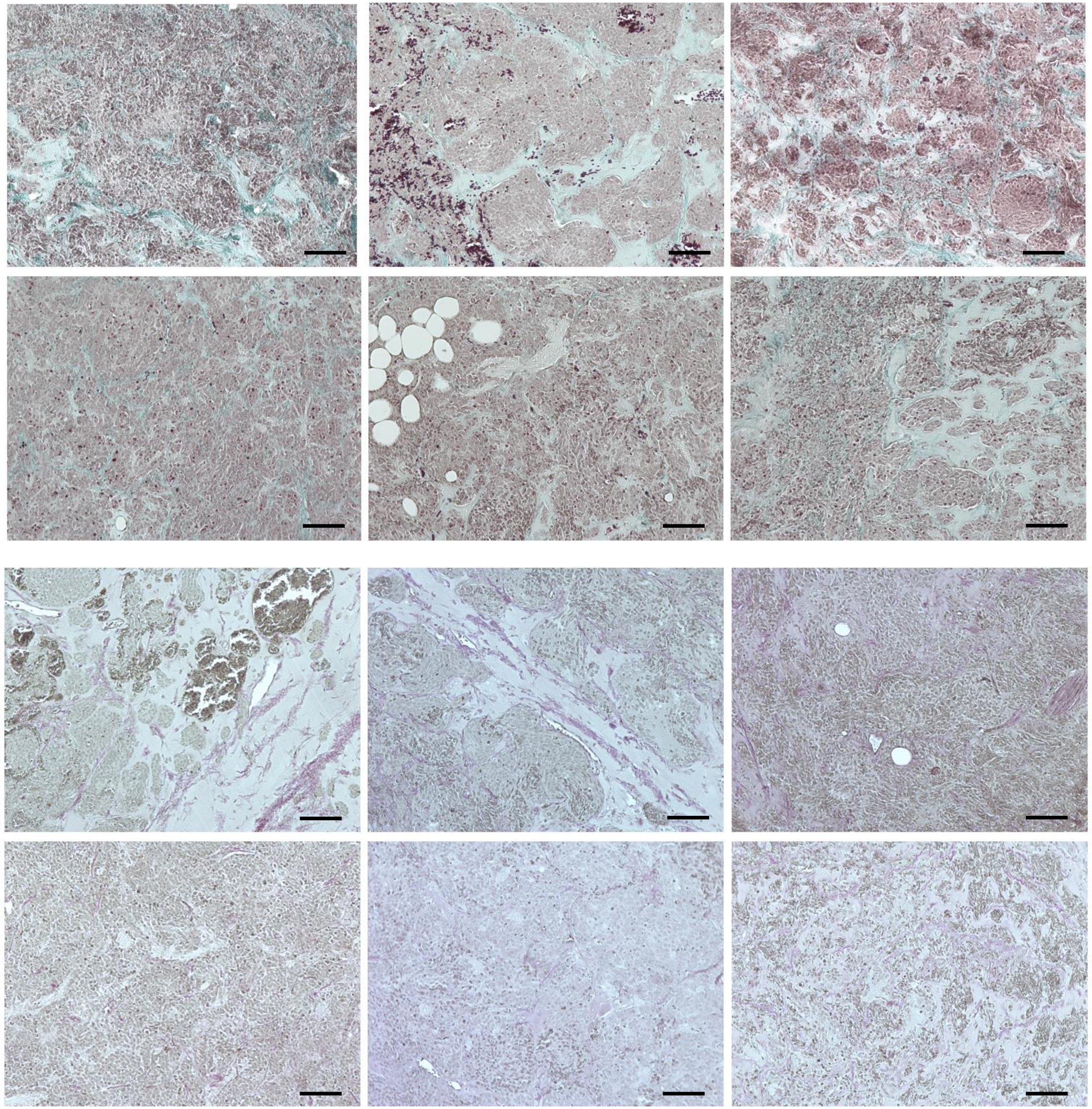

Masson-Goldner staining

shP — Mouse 1316, 1310, 1314, respectively

shC — Mouse 1302, 1303, 1304, respectively

van Gieson staining

shP — Mouse 1311, 1312, 1313, respectively

shC — Mouse 1302, 1306, 1307, respectively

Microscopic images of slices of xenograft neuroblastoma tumors grown from *PHLDA1*-silenced (shP) and control (shC) IMR-32 cells with collagen stainings. Six different tumors were presented for each type of staining. Scale bars – 100 µm.

Fig. S19. Anti-cleaved PARP-1 staining - additional microphotographs than presented in Fig. 3c

cleaved PARP-1 shP

cleaved PARP-1 shC

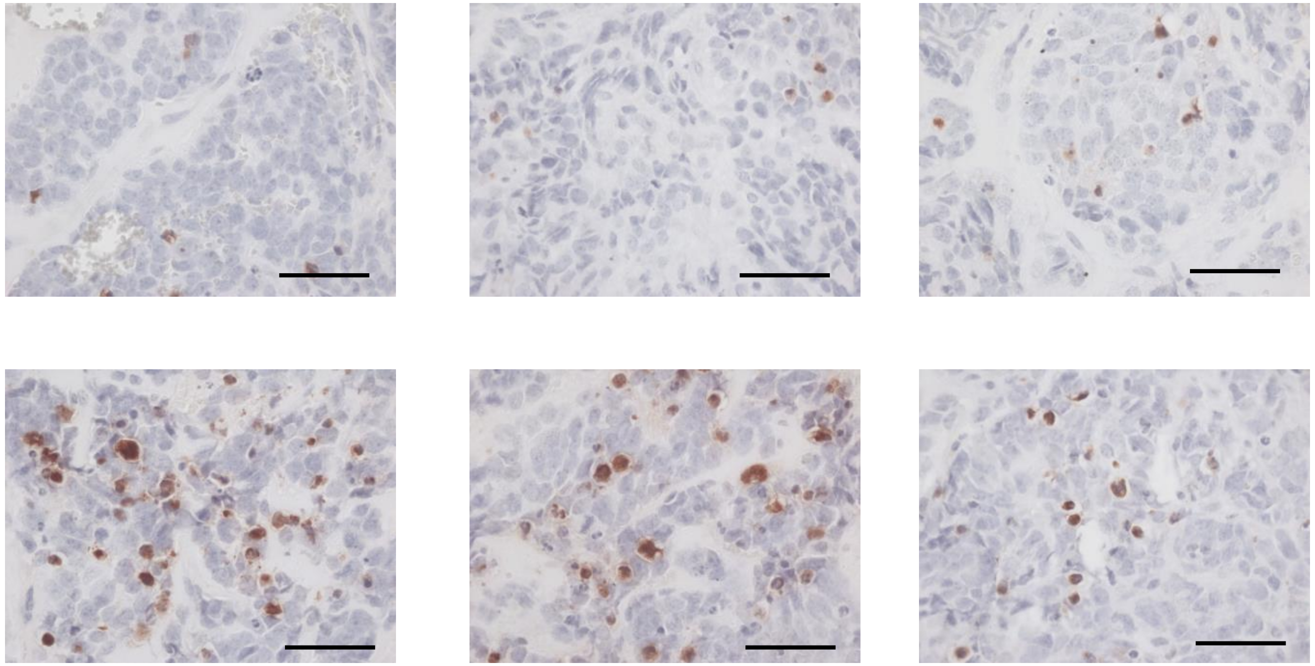

Mouse 1311, 1312, 1314, respectively

Mouse 1304, 1305, 1308, respectively

Microscopic images of slices of xenograft neuroblastoma tumors grown from *PHLDA1*-silenced (shP) and control (shC) IMR-32 cells stained with anti-cleaved PARP-1 antibodies. Scale bars – 100 µm.

Fig. S20. Anti-cleaved caspase-3 staining - additional microphotographs than presented in Fig. 3c

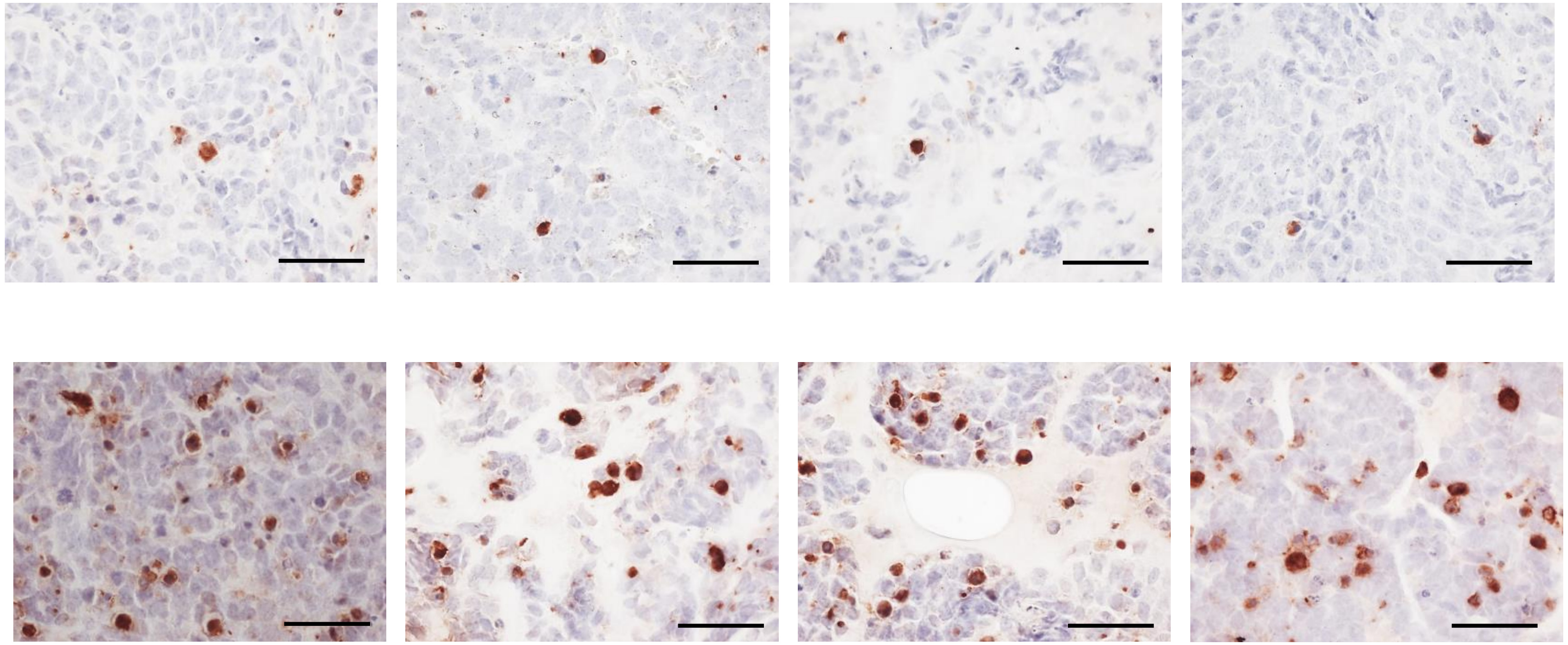

cleaved caspase-3
shP

Mouse 779, 1311, 1312, 1315, respectively

cleaved caspase-3
shC

Mouse 1302, 1304, 1305, 1308, respectively

Microscopic images of slices of xenograft neuroblastoma tumors grown from *PHLDA1*-silenced (shP) and control (shC) IMR-32 cells stained with anti-cleaved caspase-3 antibodies. Scale bars – 100 µm.

Fig. S21. Anti-PHLDA1 staining - additional microphotographs than presented in Fig. 5a

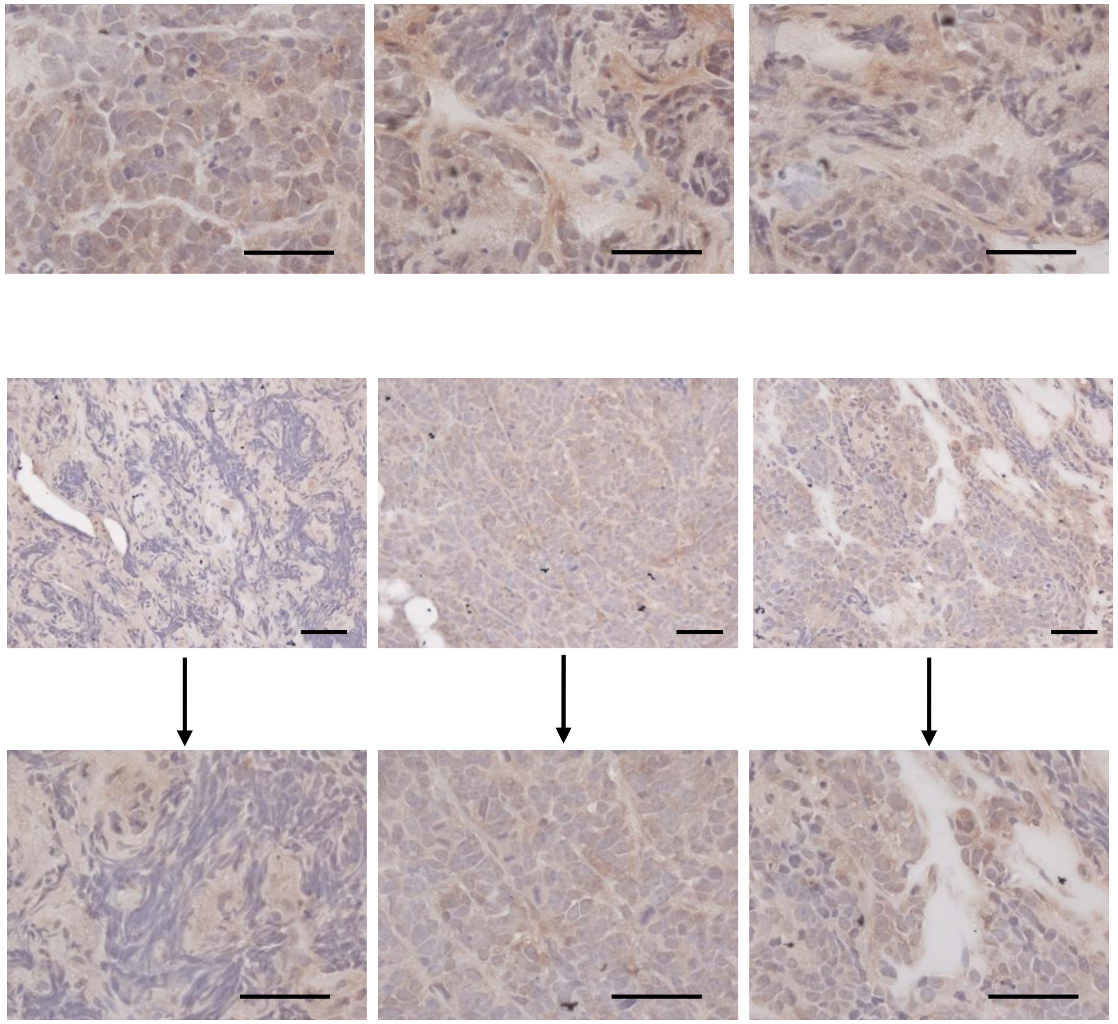

PHLDA1 shC

Three regions of one shC tumor presented.
Mouse no. 1306.

Scale bars – 100 µm

PHLDA1 shP

Same regions, different magnifications.
Three regions of one shP tumor presented.
Mouse no. 1309.

Scale bars – 100 µm

Scale bars – 100 µm

Microscopic images of slices of xenograft neuroblastoma tumors grown from *PHLDA1*-silenced (shP) and control (shC) IMR-32 cells stained with anti-PHLDA1 antibodies.

Fig. S22. Anti-ABCB1 staining - additional microphotographs than presented in Fig. 5a

ABCB1 shC

Five regions of one shC tumor presented.
Mouse no. 1306.

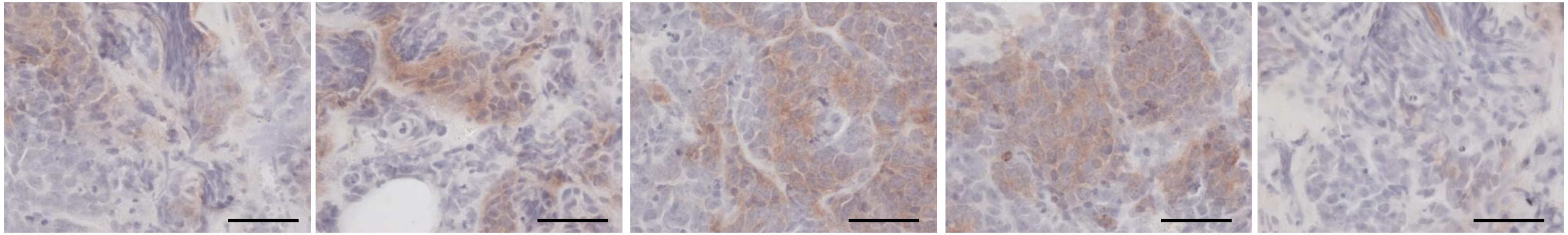

Scale bars – 100 µm

ABCB1 shP

Same regions, different magnifications. Three regions of one shP tumor presented.
Mouse no. 1309.

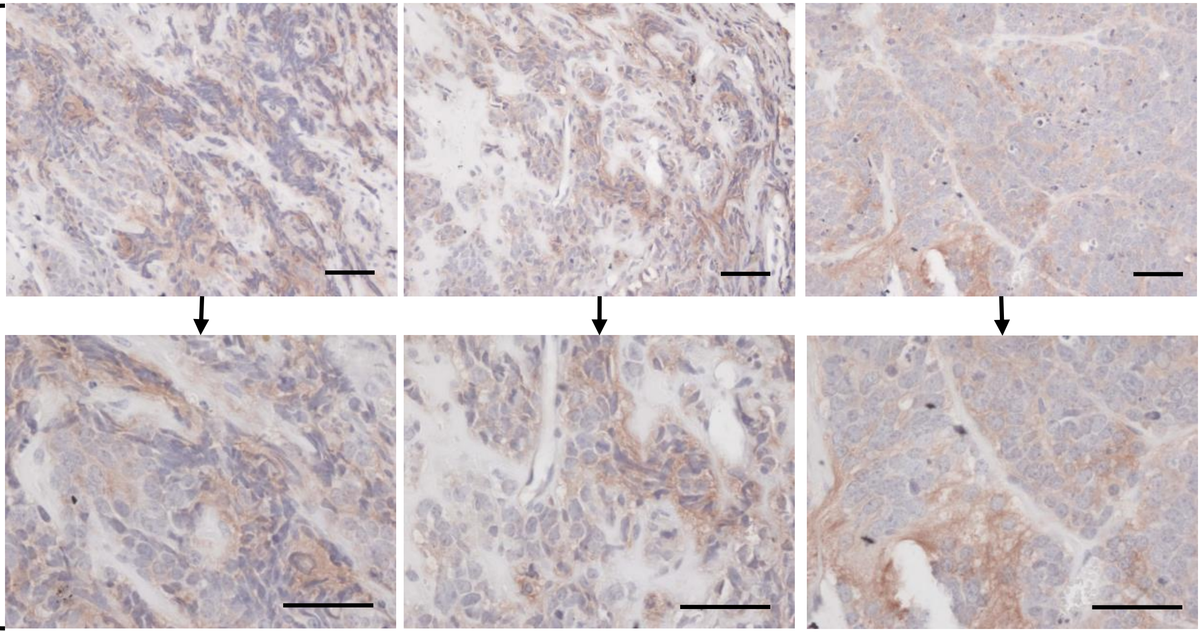

Scale bars – 100 µm

Scale bars – 100 µm

Microscopic images of slices of xenograft neuroblastoma tumors grown from *PHLDA1*-silenced (shP) and control (shC) IMR-32 cells stained with anti-ABCB1 antibodies.

Images of the positive control for anti-ABCB1 antibody - mouse kidney

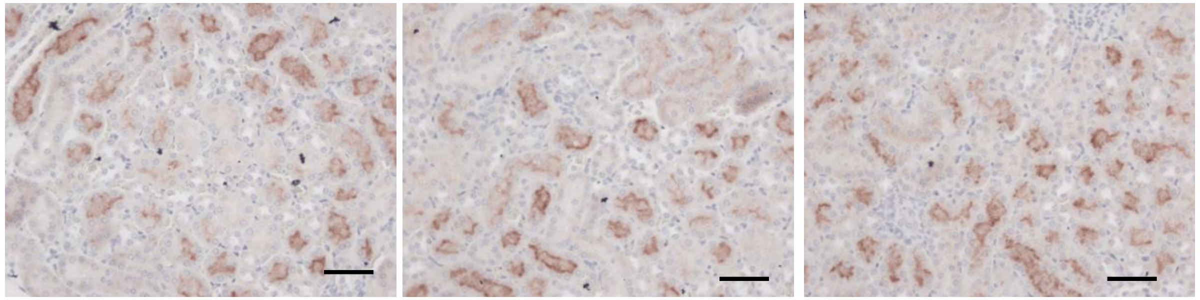

Scale bars – 100 µm

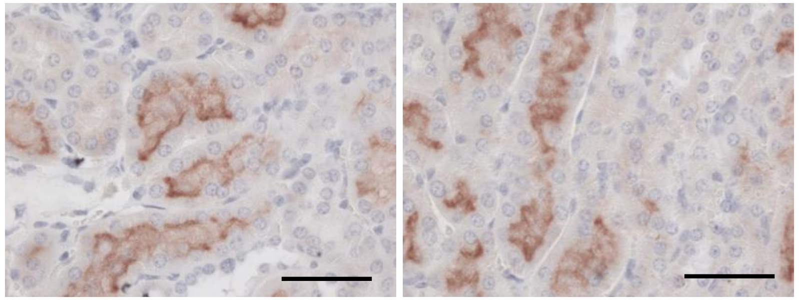

Scale bars – 100 µm

Fig. S23. The overall survival rate of neuroblastoma patients correlated with the PCOLCE mRNA expression

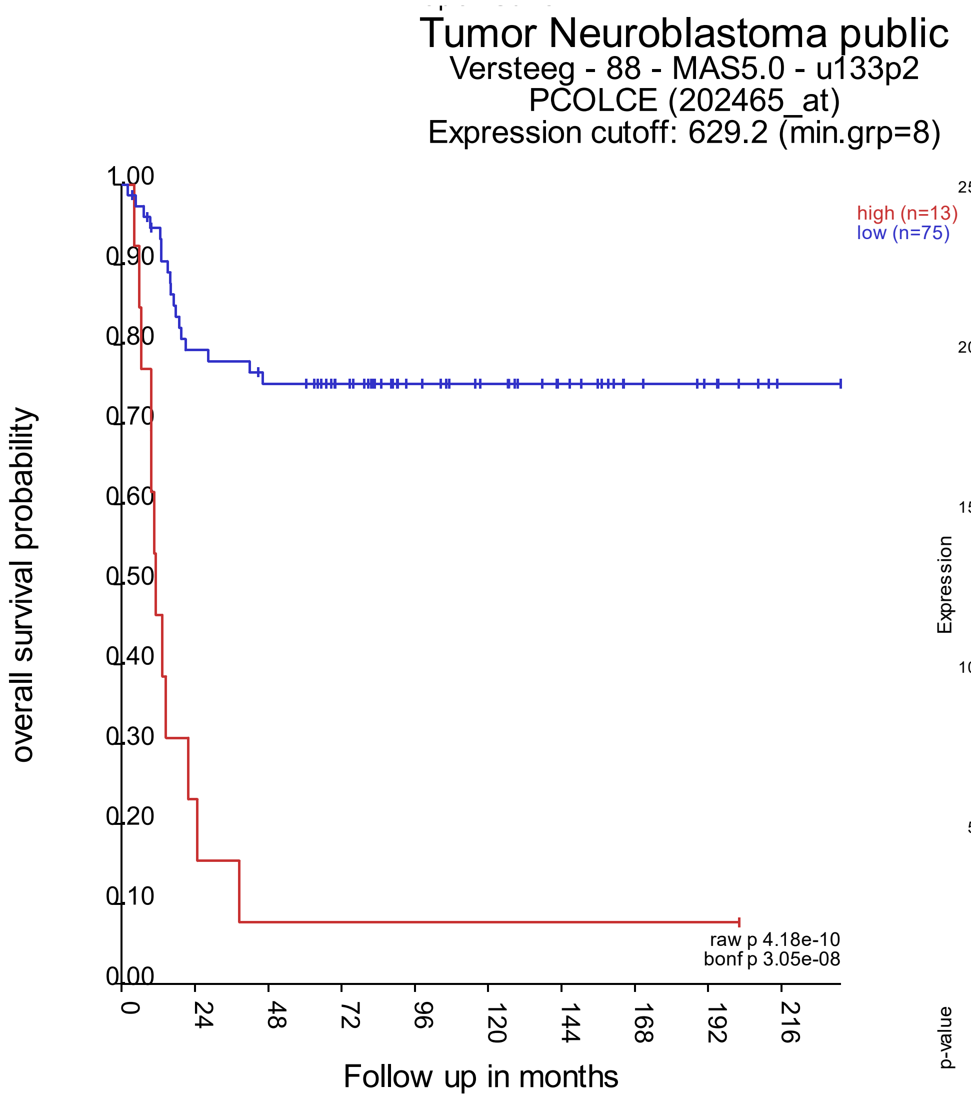

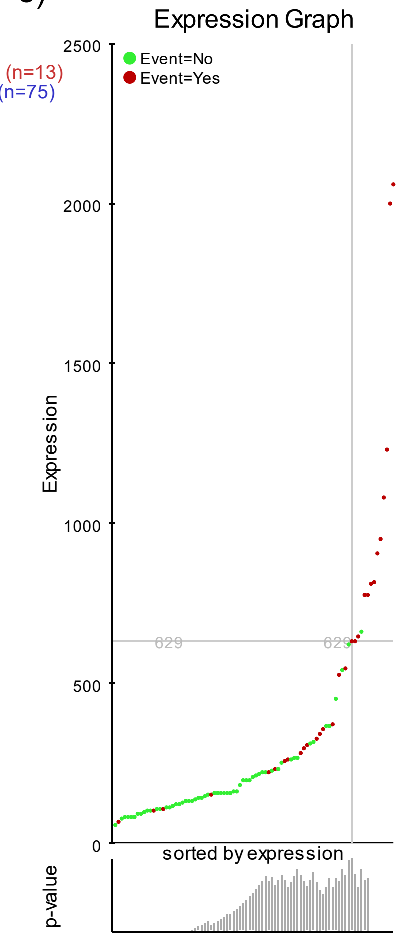

The overall survival rate of neuroblastoma patients correlated with the PCOLCE mRNA expression. Bioinformatic analysis was performed by using tools implemented in the R2 platform (http://r2.amc.nl, http://r2platform.com). The Kaplan-Meier graph correlating expression of PCOLCE mRNA with overall survival probability is shown, for samples of neuroblastoma patients from the Versteeg 88 dataset available on R2 platform.

Fig. S24. Relapse-free survival rate of neuroblastoma patients correlated with PCOLCE mRNA expression

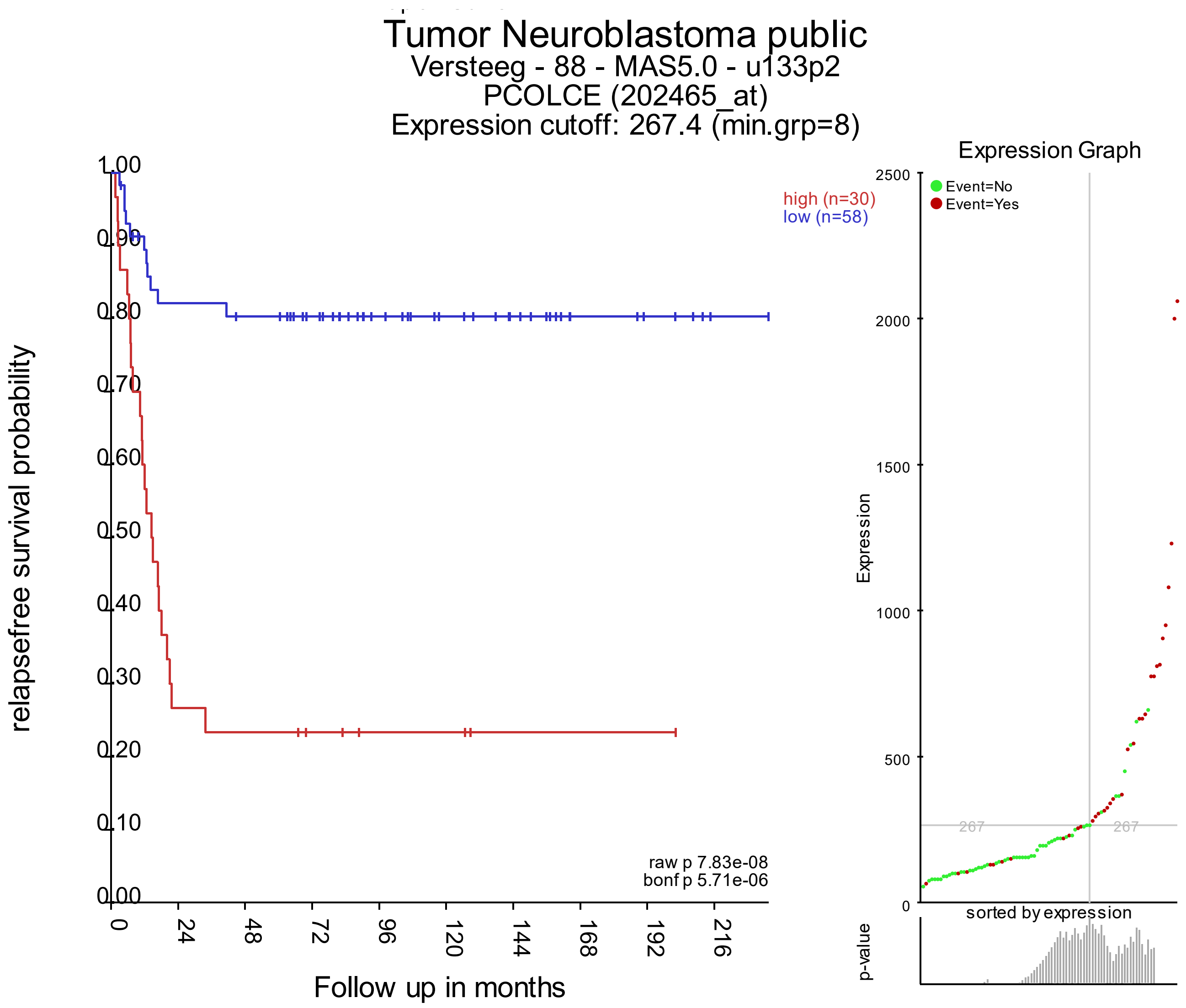

The relapse-free survival of neuroblastoma patients correlated with the PCOLCE mRNA expression. Bioinformatic analysis was performed by using tools implemented in the R2 platform (http://r2.amc.nl, http://r2platform.com). The Kaplan-Meier graph correlating expression of PCOLCE mRNA with relapse-free survival probability is shown, for samples of neuroblastoma patients from the Versteeg 88 dataset available on R2 platform.

Table S1a. Mouse coding and tumor weights from the experiment 1 (for the mean value ±SEM presented in Fig. 3b).

Group	Mouse no.	Tumor weight [g]
shC	1301	0.07
shC	1302	0.184
shC	1303	0.127
shC	1304	0.14
shC	1305	0.067
shC	1306	0.216
shC	1307	0.09
shC	1308	0.108
shP	1309	0.42
shP	1310	0.309
shP	1311	0.23
shP	1312	0.294
shP	1313	0.196
shP	1314	0.304
shP	1315	0.339
shP	1316	0.23

shP – *PHLDA1*-silenced IMR-32 cells
shC – control IMR-32 cells

Table S1b. Mouse coding and tumor weights from the experiment 2 (for the mean value ±SEM presented in Fig. 3b).

Group	Mouse no.	Tumor weight [g]
shC	770	0.236
shC	771	0.214
shC	772	0.036
shC	773	0.048
shC	774	0.197
shC	775	0.046
shC	776	0.058
shC	777	0.167
shP	778	0.076
shP	779	0.283
shP	780	0.429
shP	781	0.161
shP	782	0.179
shP	783	0.193
shP	784	0.193
shP	785	0.24

Table S2a. Results of counting of apoptotic cells in HE stained microphotographs of shC tumors (for the mean value ±SEM presented in Fig. 3d).

shC

Mouse no.	Apoptotic cells per 5 observed fields				
1308	9	8	13	5	9
1303	11	6	6	3	9
1304	11	14	13	8	6
1306	7	8	9	6	7
1302	9	5	12	13	10
1305	4	5	6	7	7

Table S2b. Results of counting of apoptotic cells in in HE stained microphotographs of shP tumors (for the mean value ±SEM presented in Fig. 3d).

shP

Mouse no.	Apoptotic cells per 5 observed fields				
1316	4	5	10	11	3
1313	12	12	8	4	2
1310	3	5	11	12	7
1311	10	16	3	7	4
1312	10	2	5	5	5
1315	4	6	8	4	4
1314	7	6	7	5	2

shP – *PHLDA1*-silenced IMR-32 cells
shC – control IMR-32 cells

Table S2c. Results of counting of PARP-1-positive apoptotic cells in microphotographs of shC tumors (for the mean value ±SEM presented in Fig. 3d).

shC

Mouse no.	Cleaved PARP-1-positive cells per 5 observed fields				
1308	45	38	21	30	24
1303	59	58	46	40	43
1304	65	58	61	66	48
1306	39	29	29	28	31
1302	47	49	37	28	38
1307	30	27	28	32	32
1305	48	47	62	51	69
1309	65	59	58	49	59
771	51	45	53	32	32

Table S2d. Results of counting of PARP-1-positive apoptotic cells in microphotographs of shP tumors (for the mean value ±SEM presented in Fig. 3d).

shP

Mouse no.	Cleaved PARP-1-positive cells per 5 observed fields				
1316	36	27	23	28	26
1313	16	10	15	11	11
1310	30	10	19	36	20
1311	30	27	23	27	29
1312	15	15	15	23	19
1315	12	9	5	14	15
1314	14	21	5	15	13
779	28	36	32	30	38
785	26	38	28	41	31

Table S2e. Results of counting of cleaved caspase 3-positive apoptotic cells in microphotographs of shC tumors (for the mean value ±SEM presented in Fig. 3d).

shC

Mouse no.	Cleaved caspase 3-positive cells per 5 observed fields				
1308	58	62	76	66	54
1303	24	39	22	26	22
1304	49	37	48	37	31
1306	29	15	36	35	20
1302	31	36	28	67	37
1307	28	22	25	18	19
1305	46	47	48	49	41
1309	16	20	22	25	19
771	38	20	34	36	18

Table S2f. Results of counting of cleaved caspase 3-positive apoptotic cells in microphotographs of shP tumors (for the mean value ±SEM presented in Fig. 3d).

shP

Mouse no.	Cleaved caspase 3-positive cells per 5 observed fields				
1316	18	12	22	15	14
1313	16	15	16	18	16
1310	4	13	13	8	8
1311	23	12	18	9	16
1312	12	23	14	22	17
1315	3	2	0	4	4
1314	8	5	7	5	6
779	14	8	17	22	14
785	16	16	21	20	12

Table S3. Results of counting of PHLDA1-positive and ABCB1-positive cells in microphotographs of shP and shC tumors (for the mean value ±SEM presented in Fig. 5b).

Mouse no.	Group	PHLDA1-positive cells [% x staining intensity]	ABCB1-positive cells [% x staining intensity]
785	shP	9.5	45
779	shP	20	30
1311	shP	7.5	18
1315	shP	15	7.5
775	shC	9.5	20.5
771	shC	40	7.5
1309	shC	25	20
1306	shC	35	17.5

shP – *PHLDA1*-silenced IMR-32 cells
shC – control IMR-32 cells
